# Harnessing work-function-driven rotational steering for quantum state control in HCl dissociation on bimetallic alloys

**DOI:** 10.1039/d6sc00201c

**Published:** 2026-02-04

**Authors:** Tianhui Liu, Kaixin Meng

**Affiliations:** a School of Sciences, Great Bay University Dongguan 523000 China liutianhui@gbu.edu.cn; b Great Bay Institute for Advanced Study Dongguan 523000 China; c State Key Laboratory of Chemical Reaction Dynamics, Dalian Institute of Chemical Physics, Chinese Academy of Sciences Dalian 116023 China

## Abstract

The dissociative chemisorption of heteronuclear molecules is a cornerstone of heterogeneous catalysis. However, the ability to predict and control how rotational excitation governs reactivity has remained a fundamental challenge, lagging far behind the established understanding of vibrational effects. Here, through six-dimensional quantum dynamics simulations of HCl dissociation on bimetallic surfaces, we report unprecedented rotational enhancement, with efficacies reaching roughly 225 and 56 on Ag/Pt(111) and Cu/Pt(111), respectively. This dramatic effect originates from interfacial charge transfer driven by work function differences between the substrate and the supported metal monolayer (*Φ*_sub_ > *Φ*_sup_), which generates a non-monotonic orientation-dependent potential energy landscape. We further establish a quantitative, predictive design principle, where rotational efficacy scales primarily with the work function difference and is systematically modulated by surface strain, with the highest efficacy achieved when a large work function difference is combined with compressive strain. This multivariate framework resolves a long standing dichotomy by demonstrating that rotational effects depend decisively on the global topography of the potential energy surface (PES), a mechanism fundamentally distinct from the transition state (TS) localized picture of vibrational promotion. The resulting quantum state control window enables rotation to act as a precise external knob for steering reactivity, advancing a new paradigm for the design of catalysts with targeted, state selective function.

## Abstract 2

The dissociative chemisorption of heteronuclear molecules is a cornerstone of heterogeneous catalysis.

## Graphical abstract

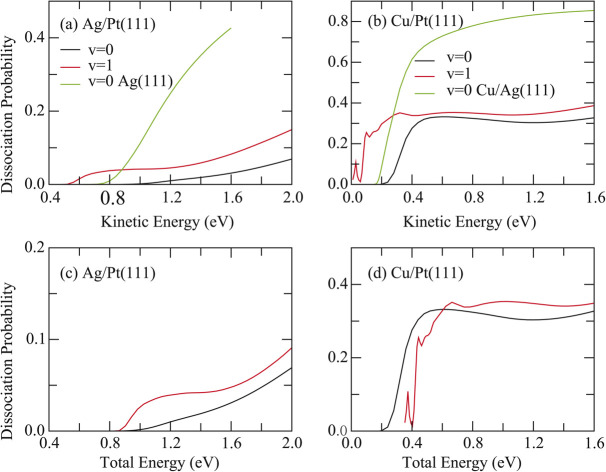

The dissociative chemisorption of gas-phase molecules on transition-metal surfaces constitutes a critical elementary step in heterogeneous catalysis, frequently governing reaction rates in pivotal industrial processes such as ammonia synthesis, hydrocarbon reforming, and hydrogen production. Over decades, concerted experimental and theoretical efforts have elucidated the complex energy landscapes underlying these surface reactions, with particular emphasis on the manner in which energy partitioning among translational, vibrational, and rotational degrees of freedom modulates reactivity.^1–42^

Recent advances in quantum-state-resolved laser techniques have enabled precise preparation of reactant molecules in specific rovibrational states, permitting detailed examination of the respective contributions of the distinct forms of molecular energy to chemical reactivity. Concurrent developments in theoretical methodologies, including quasi-classical trajectory (QCT) calculations and quantum dynamical simulations on high-dimensional potential energy surfaces (PESs), have achieved remarkable agreement with experimental observations.^11,19,34,42^ Furthermore, two fundamental principles emerging from these studies, *i.e.*, Polanyi's rules^43^ and the sudden vector projection (SVP) model,^44^ provide powerful predictive frameworks for understanding dynamical outcomes without requiring exhaustive dynamical calculations on global PESs.

Originally formulated for gas-phase atom–diatom reactions, Polanyi's rules have been successfully extended to gas-surface systems. These empirical guidelines propose that reactions characterized by reactant-like (“early”) transition states (TSs) preferentially utilize translational energy to overcome the barrier, whereas those with product-like (“late”) TSs benefit more effectively from vibrational excitation.^43^ To address limitations in predicting mode-specific and bond-selective behavior for polyatomic systems, the SVP model was subsequently developed and adapted for gas-surface reactions.^44^ This model quantifies the alignment between reactant vibrational modes and the reaction coordinate at the TS. Together, these frameworks facilitate rapid assessment of vibrational efficacy observed in numerous systems. In many cases, vibrational energy proves more efficient than translational energy in promoting bond cleavage, resulting in a vibrational efficacy exceeding unity.^19,21,35,39^

In contrast to the well-established role of vibrational excitation, rotational energy has consistently been observed to exert negligible or inhibitory effects on reactivity across many prototypical systems, such as H_2_ on the Cu(111) and Ag(111), D_2_O on Ni(111), and CH_4_ on Ni(100) surfaces.^1,2,5,14,30,32^ For instance, studies of H_2_ dissociation on Cu(111) revealed rotational efficacy values invariably below unity, signifying that rotational energy is less effective than translational energy in enhancing reactivity.^1,2^ Furthermore, these systems consistently demonstrate preferential reactivity for molecules in helicopter alignment (rotation parallel to the surface) compared to cartwheel alignment (rotation perpendicular to the surface).^3,18,24,26–29,31,32^ This persistent alignment preference appears particularly intriguing given the substantial variation in TS geometries across different reactions, ranging from parallel H–H bond orientations (90°) in H_2_ dissociation to tilted C–H bond configurations (roughly 45°) in CH_4_ dissociation. The strong preference for helicopter alignment in CH_4_ dissociation on Ni(100) was attributed by Jiang *et al.* to the overlap between the initial molecular alignment and the transition state geometry, as demonstrated through QCT calculations on a twelve-dimensional global PES.^31^

This conventional understanding has been fundamentally challenged by recent breakthroughs. Gerrits *et al.* reported a striking rotational enhancement, exceeding an order of magnitude (20-fold), in HCl dissociation on Au(111),^35^ which is correlated with a non-monotonic dependence of the minimum energy path (MEP) on molecular orientation. Subsequent work by our group revealed even greater rotational efficacy (roughly 90) for HCl dissociation on the Ag/Au(111) bimetallic alloy surface.^39^ Density functional theory (DFT) calculations demonstrated that Ag–Au interfacial charge transfer modifies the PES topography, creating a more pronounced non-monotonic angular (*θ*) dependence of the MEP.^37,39^ Remarkably, this modified angular landscape induced an inversion of rotational alignment preference, where dissociation of low-*j* state (*j* = 1) favored cartwheel alignment (*m* = 0) while high-*j* state (*j* = 5) preferentially dissociated *via* helicopter alignment (*m* = *j*).^39^ Analogous phenomena were observed for HCl dissociation on Cu/Au(111),^41^ suggesting that bimetallic interfaces may universally enable rotational steering through controlled electronic modifications.

In this Letter, the quantum dynamics of HCl dissociation are explored on Ag/Pt(111) and Cu/Pt(111) bimetallic alloy surfaces, formed by the deposition of strained pseudomorphic monolayers of Ag and Cu onto a Pt(111) substrate, respectively. Given the comparable work functions of Pt and Au, this work examines whether the rotational enhancement phenomena observed in Ag/Au(111) and Cu/Au(111) systems persist in these alternative charge-transfer-dominated bimetallic combinations. Through systematically probing rotational excitation and alignment effects on HCl dissociation across a series of bimetallic alloys, the ultimate objective is to advance a generalized model for engineering rotational energy landscapes *via* interfacial electronic control. This framework ultimately seeks to establish principles for rotational manipulation in gas-surface reactions and enable catalyst designs where molecular rotation serves as a precise control parameter.

The reaction dynamics were investigated through six-dimensional (6D) time-dependent wave packet (TDWP) calculations performed on two globally accurate PESs constructed *via* neural network (NN) fitting. These PESs were parameterized using approximately 43 000 and 30 000 DFT energy points for the HCl + Ag/Pt(111) and HCl + Cu/Pt(111) systems, respectively, yielding root-mean-square errors (RMSEs) of 4.4 meV and 3.7 meV. Static barrier heights of 0.95 eV and 0.30 eV were determined for HCl dissociation on Ag/Pt(111) and Cu/Pt(111) surfaces (Fig. S2), exhibiting substantial deviations from the values obtained for pure Ag(111) (0.85 eV) and Cu(111) (0.52 eV) surfaces. These differences were primarily attributed to surface strain effects, where the Ag monolayer on Ag/Pt(111) undergoes 5.7% compression while the Cu monolayer on Cu/Pt(111) experiences 8.0% expansion. These observations are consistent with established strain–reactivity correlations, where compressive strain typically inhibits reactivity whereas expansive strain enhances it.^37,40,41,45–48^ Additionally, ligand effects from the Pt substrate further modulate reactivity, as demonstrated by the HCl + Cu/Ag(111) system, where 14.9% Cu monolayer expansion corresponds to a 0.32 eV barrier (0.02 eV higher than HCl + Cu/Pt(111)).^41^ Further details regarding PES construction and TDWP calculations are provided in the SI.

The 6D dissociation probabilities for HCl initially in the vibrational ground state (*v* = 0, *j* = 0) and the first excited vibrational state (*v* = 1, *j* = 0) were computed for both surfaces and compared with previous results for HCl + Ag(111) and HCl + Cu/Ag(111) systems.^41,49^ Significant reactivity suppression was observed, with HCl reactivity in both non-rotating states (*v* = 0, 1) being markedly inhibited on Ag/Pt(111) and Cu/Pt(111) surfaces. As shown in Fig. 1(a), the 6D dissociation probability of HCl in the (*v* = 0) state on Ag/Pt(111) is substantially lower than that on Ag(111), despite only a 0.10 eV higher barrier. For instance, at a kinetic energy of 1.6 eV, the probabilities for *v* = 0 HCl on Ag/Pt(111) and Ag(111) surfaces are 0.0308 and 0.427, respectively. Furthermore, vibrational excitation (*v* = 1) yields a probability of 0.0826 at this energy, still significantly below the *v* = 0 value on Ag(111).

**Fig. 1:**
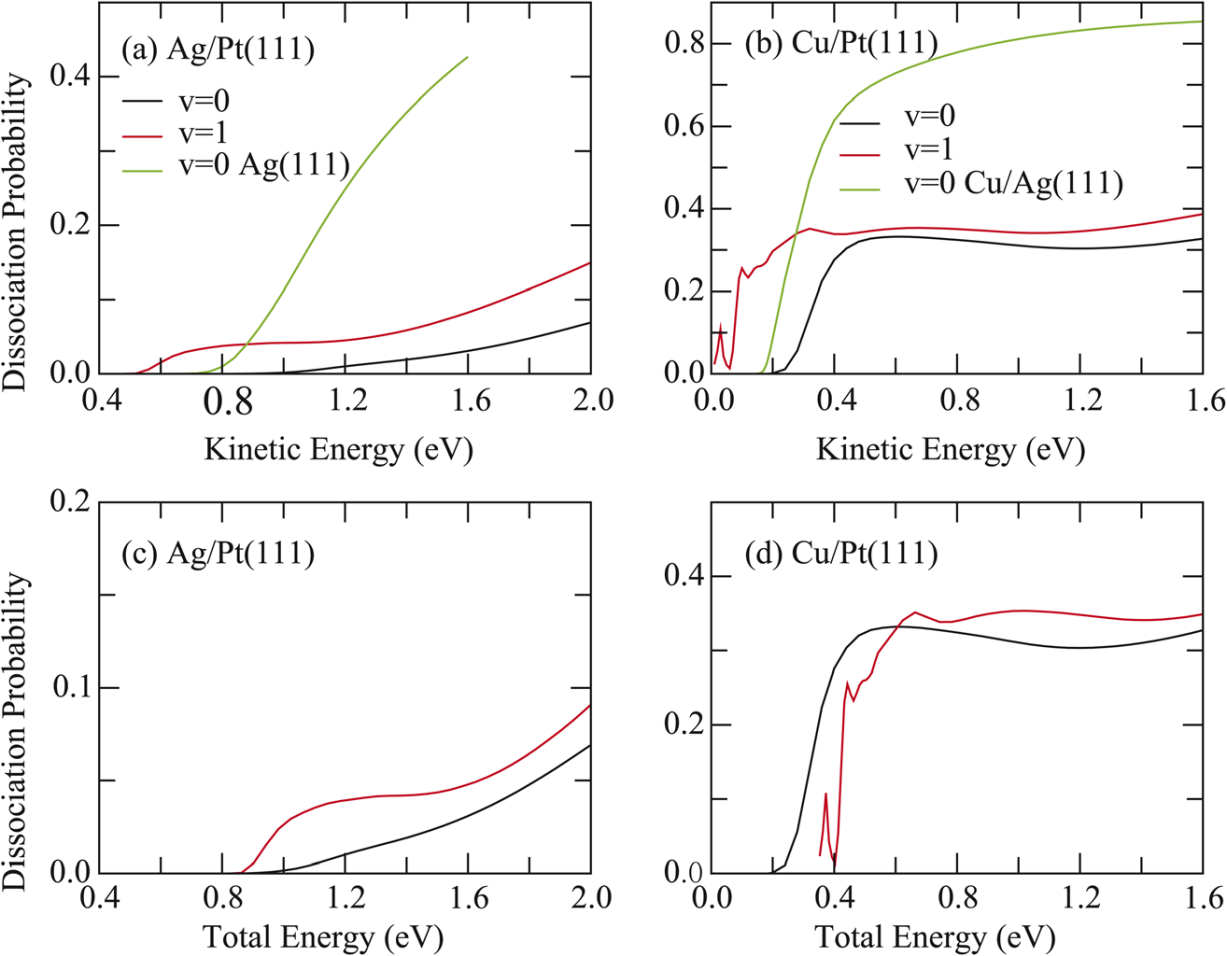
(a) Comparison of the 6D dissociation probabilities as a function of kinetic energy with HCl initially in the two non-rotating states (*v* = 0, 1) on Ag/Pt(111) and HCl initially in *v* = 0 on Ag(111). (b) The same as in (a), except for the comparison between Cu/Pt(111) and Cu/Ag(111) surfaces. (c) Comparison of the 6D dissociation probabilities as a function of total energy with HCl initially in the two non-rotating states (*v* = 0, 1) on Ag/Pt(111). (d) The same as in (c), except for HCl dissociation on the Cu/Pt(111) surface.

Similar behavior was observed for HCl + Cu/Pt(111) (Fig. 1(b)). Despite a 0.02 eV lower barrier compared to the HCl + Cu/Ag(111) reaction, the 6D probabilities for HCl (*v* = 0) across the entire energy range and for HCl (*v* = 1) at energies above 0.28 eV on Cu/Pt(111) were consistently reduced relative to those of HCl (*v* = 0) on Cu/Ag(111). This is evidenced by comparison of the probability values, which are 0.327, 0.387 for HCl (*v* = 0, 1) on Cu/Pt(111) and 0.854 for HCl (*v* = 0) on Cu/Ag(111) at a kinetic energy of 1.6 eV, respectively.

Fig. 1(c) and (d) present the 6D dissociation probabilities for HCl (*v* = 0, 1) on both surfaces as a function of total energy, referenced to the asymptotic energy of the ground rovibrational state of HCl. For HCl + Ag/Pt(111), the dissociation probabilities for *v* = 1 systematically exceed values for *v* = 0, confirming that vibrational energy promotes dissociation more effectively than translational energy. This observation aligns with Polanyi's rules for this late-barrier reaction (*r* = 3.58 bohr at the TS). In contrast, for HCl + Cu/Pt(111), the dissociation probabilities for *v* = 1 are lower than those for *v* = 0 below 0.62 eV but become slightly higher above this energy. This trend can be attributed to its earlier barrier (*r* = 3.08 bohr), lower barrier height, and looser saddle point, compared to the HCl + Ag/Pt(111) reaction (shown in Fig. S4(a)–(d)).

Fig. 2(a) shows the averaged 6D dissociation probabilities (*P*^average^_*v*,*j*_) for HCl initially in the vibrational ground state (*v* = 0) with rotational states ranging from *j* = 0 to 8 on Ag/Pt(111) as a function of kinetic energy. The quantity *P*^average^_*v*,*j*_ was obtained by averaging over *P*_*v*,*j*_^*m*^, where *P*_*v*,*j*_^*m*^ represents the dissociation probability from the initial state (*v*, *j*, *m*) of HCl. All rotationally excited HCl states exhibit significantly higher reactivity than the rovibrational ground state, demonstrating that rotational excitation of HCl substantially enhances reactivity. The dissociation probability *P*^average^_0,*j*_ increases with *j* from 1 to 5, although *P*^average^_0,5_ is slightly smaller than *P*^average^_0,4_ at energies below 1.17 eV. At higher rotational states (*j* = 6–8), *P*^average^_0,*j*_ increases at low kinetic energies, though *P*^average^_0,7_ becomes the largest and *P*^average^_0,8_ the smallest at high kinetic energies. When examined as a function of the total energy (Fig. 2(b)), *P*^average^_0,1–8_ consistently exceeds the corresponding value for HCl (*v* = 0, *j* = 0) across the entire energy region. Notably, *P*^average^_0,1–8_ also surpasses the corresponding value for HCl (*v* = 1, *j* = 0) at higher total energies. For example, *P*^average^_0,1_ exceeds the *v* = 1 value when the total energy exceeds 1.28 eV.

**Fig. 2:**
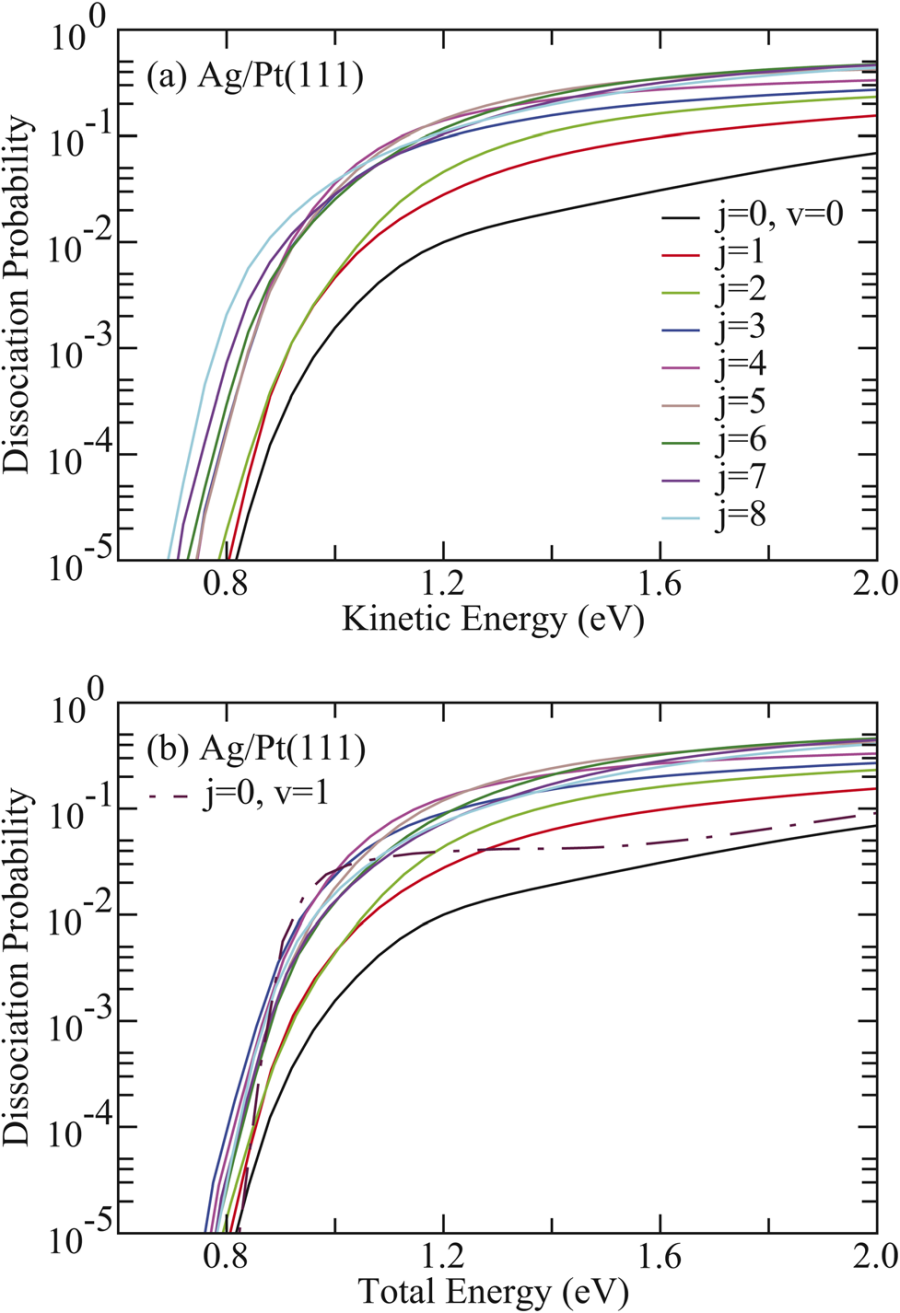
(a) Comparison of the 6D dissociation probabilities as a function of kinetic energy with HCl initially in the rotational states (*v* = 0, *j* = 1–8) on Ag/Pt(111). (b) Same as in (a), except as a function of total energy. The results with HCl initially in the vibrational excited state (*v* = 1, *j* = 0) are also included in (b) for comparison.

The corresponding results for HCl on Cu/Pt(111) are illustrated in Fig. 3(a) and (b). Despite the 0.65 eV lower barrier height compared to HCl + Ag/Pt(111), the HCl + Cu/Pt(111) system exhibits analogous rotational enhancement effects. Given the relatively weak vibrational excitation effect for HCl + Cu/Pt(111), *P*^average^_0,1–8_ consistently exceeds the corresponding value for HCl (*v* = 1, *j* = 0) across the total energy region (Fig. 3(b)). However, *P*^average^_0,5–8_ becomes smaller than the value for HCl (*v* = 0, *j* = 0) at low total energies. For instance, *P*^average^_0,8_ underestimates the *v* = 0 value when total energies are below 0.44 eV.

**Fig. 3:**
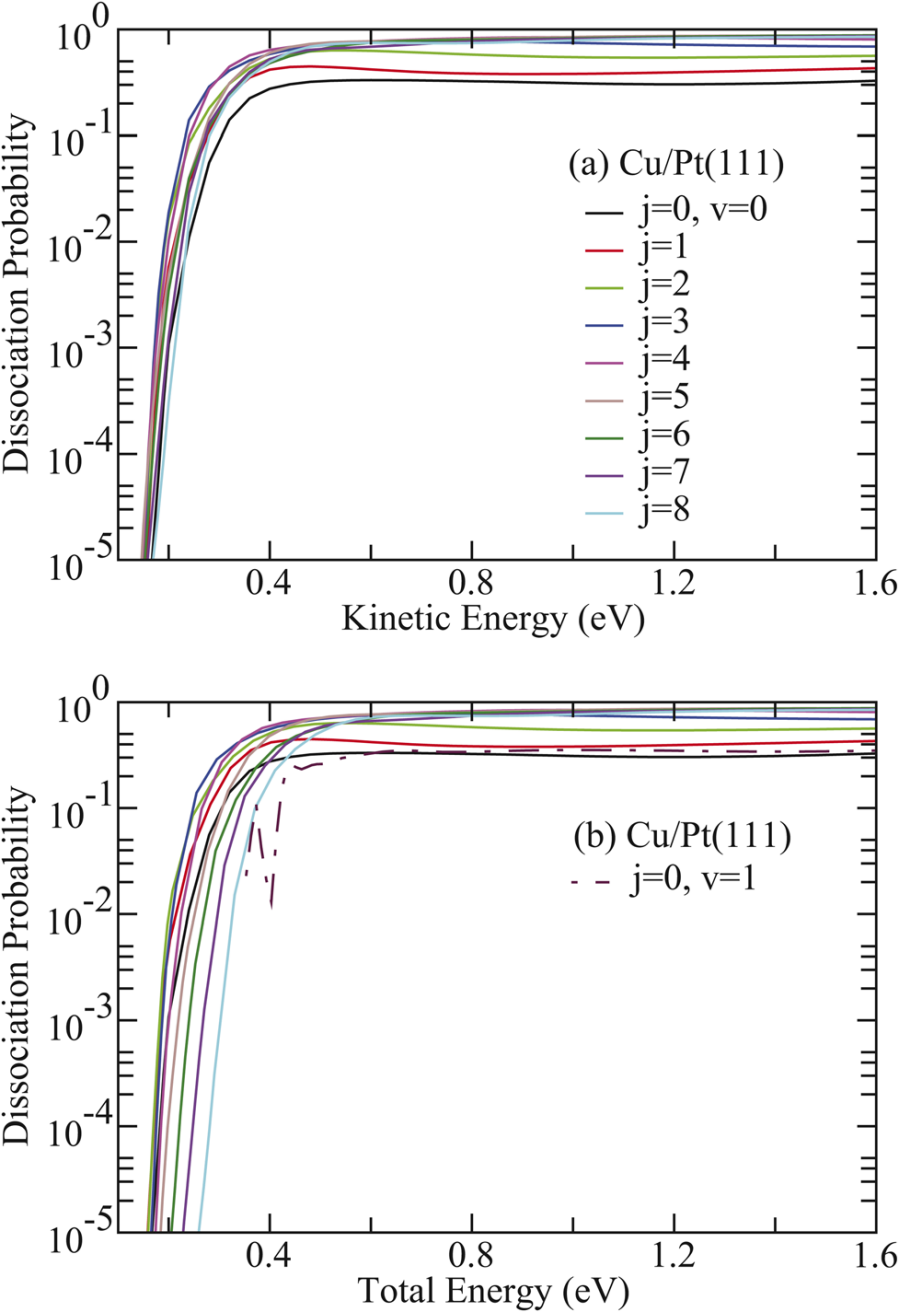
(a) Comparison of the 6D dissociation probabilities as a function of kinetic energy with HCl initially in the rotational states (*v* = 0, *j* = 1–8) on Cu/Pt(111). (b) Same as in (a), except as a function of total energy. The results with HCl initially in the vibrational excited state (*v* = 1, *j* = 0) are also included in (b) for comparison.

These observations underscore the superior efficacy of rotational excitation in promoting HCl dissociation compared to equivalent amounts of translational or vibrational energy. This conclusion is quantitatively supported by rotational efficacy values presented in Table 1 and 2 for HCl dissociation on Ag/Pt(111) and Cu/Pt(111) surfaces, respectively. For HCl + Ag/Pt(111), all rotational efficacy values significantly exceed unity and substantially surpass the corresponding vibrational efficacy values at equivalent dissociation probabilities. Most remarkably, the HCl (*v* = 0, *j* = 1) state, exhibiting the maximum probability (*P*_0_ = 0.0692), yields an unprecedented value of 224.81 (Table 1), representing the highest value reported in gas-surface reaction systems. Similarly, for HCl + Cu/Pt(111), the HCl (*v* = 0, *j* = 1) state (*P*_0_ = 0.319) demonstrates a rotational efficacy of 55.76 (Table 2). Both systems demonstrate consistent trends in which rotational efficacy exhibits a positive correlation with both dissociation probability and collision energy for specific rotational states, while decreasing systematically with increasing rotational quantum number from *j* = 1 to *j* = 8 at fixed dissociation probability *P*_0_.

**Table 1:** Rotational efficacies for the excited rotational states (*v* = 0, *j* = 1–8) and vibrational efficacies for the first excited vibrational state (*v* = 1, *j* = 0) for dissociative chemisorption of HCl on Ag/Pt(111) calculated by a full-dimensional quantum dynamical approach

Rovibrational state	Rotational/vibrational efficacy, *η*				
	*P*_0_ = 10^−5^	*P*_0_ = 10^−4^	*P*_0_ = 10^−3^	*P*_0_ = 10^−2^	*P*_0_ = 0.0692
*v* = 0, *j* = 1	3.38	10.13	22.43	54.57	224.81
*v* = 0, *j* = 2	4.30	3.9	7.61	20.42	95.58
*v* = 0, *j* = 3	5.16	6.02	8.53	18.18	57.84
*v* = 0, *j* = 4	3.02	3.58	5.09	11.16	36.75
*v* = 0, *j* = 5	2.03	2.38	3.46	7.27	24.27
*v* = 0, *j* = 6	1.64	1.95	2.76	5.18	16.78
*v* = 0, *j* = 7	1.60	1.74	2.33	4.18	12.43
*v* = 0, *j* = 8	1.39	1.62	2.18	3.62	10.02
*v* = 1, *j* = 0	0.96	1.11	1.3	1.81	1.84

**Table 2:** Rotational efficacies for the excited rotational states (*v* = 0, *j* = 1–8) and vibrational efficacies for the first excited vibrational state (*v* = 1, *j* = 0) for dissociative chemisorption of HCl on Cu/Pt(111) calculated by a full-dimensional quantum dynamical approach

Rovibrational state	Rotational/vibrational efficacy, *η*					
	*P*_0_ = 10^−5^	*P*_0_ = 10^−4^	*P*_0_ = 10^−3^	*P*_0_ = 10^−2^	*P*_0_ = 10^−1^	*P*_0_ = 0.319
*v* = 0, *j* = 1	4.97	6.95	8.54	11.71	9.73	55.76
*v* = 0, *j* = 2	1.92	2.59	3.38	6.15	7.08	22.27
*v* = 0, *j* = 3	1.13	1.53	1.93	3.18	4.7	13.62
*v* = 0, *j* = 4	0.76	0.92	1.04	1.6	2.55	7.98
*v* = 0, *j* = 5	0.6	0.63	0.55	0.79	1.03	4.63
*v* = 0, *j* = 6	0.34	0.32	0.30	0.47	0.62	2.85
*v* = 0, *j* = 7	0.16	0.12	0.08	0.22	0.46	2.18
*v* = 0, *j* = 8	0.02	−0.07	−0.10	0.06	0.29	1.60
*v* = 1, *j* = 0					0.80	0.74

Given that *P*^average^_*v*,*j*_ represents the degeneracy-averaged dissociation probability over different *m*-alignments, the rotational-alignment effects for the two reactions were then investigated. As presented in Fig. 4(a)–(d) for HCl + Ag/Pt(111), a striking inversion of alignment preference with rotational quantum number *j* is observed. Specifically, for the *v* = 0, *j* = 1 state, dissociation probabilities for cartwheel alignment (*m* = 0) consistently exceed those for helicopter alignment (*m* = 1), indicating preferential dissociation when the molecular axis rotates perpendicular to the surface (Fig. 4(a)). For *j* = 3, minimal reactivity occurs at *m* = 3 while maximal probabilities at energies below 0.6 eV are achieved at *m* = 2 (Fig. 4(b)). For *j* = 5, helicopter alignment (*m* = 5) prevails below 1.2 eV, transitioning to *m* = 4 dominance at higher energies (Fig. 4(c)). The most pronounced inversion emerges for *j* = 8, where *m* = 2 maximizes reactivity below 0.9 eV, while *m* = 8 becomes preferred at higher energies above 0.97 eV, suggesting that dissociation is most favored in the helicopter alignment (Fig. 4(d)). Remarkably, identical alignment inversion behavior was observed for HCl + Cu/Pt(111) (Fig. S5). These rotational phenomena show striking parallels with previous observations for HCl dissociation on Ag/Au(111) and Cu/Au(111) surfaces,^39,41^ suggesting mechanistic commonalities despite significant static barrier differences.

**Fig. 4:**
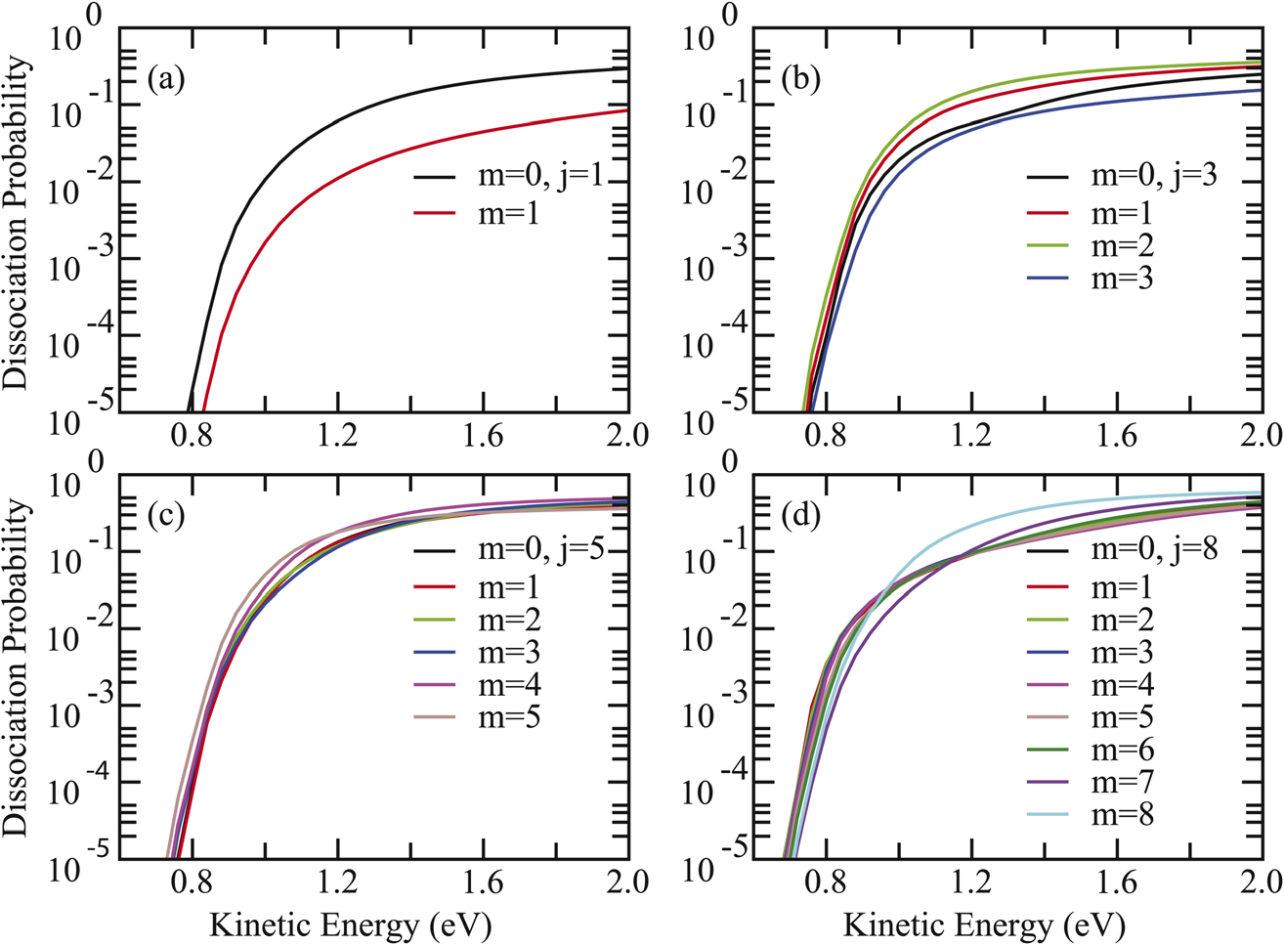
(a) The 6D dissociation probabilities with HCl initially in the same rovibrational state (*v* = 0, *j* = 1) but different alignments (*m* = 0–1) on the Ag/Pt(111) surface. (b–d) Same as (a) except for HCl initially in (*v* = 0, *j* = 3), (*v* = 0, *j* = 5), and (*v* = 0, *j* = 8) states, respectively.

The physical origin of these effects lies in the strongly non-monotonic dependence of the MEP on molecular orientation.^39^ Two-dimensional (2D) contour plots of the PES for HCl dissociation on Ag/Pt(111) are presented in Fig. 5(a) and (b), illustrating the dependence on *Z* and *θ* coordinates with *r* and *ϕ* optimized at the TS and bridge sites. Similarly, the corresponding results for HCl + Cu/Pt(111) are shown in Fig. 5(c) and (d). Despite energy differences, consistent PES topographic features are observed, where the MEP exhibits pronounced non-monotonic *θ*-dependence. Specifically, the optimal *θ* value is found to increase to angles exceeding 90° (approximately 120–150°, corresponding to Cl-down orientation) along the *Z* coordinate in the asymptotic region. It then decreases sharply to angles below 90° (approximately 40–60°, H-down orientation) in the interaction region, followed by a subsequent slight rebound before reaching the TS geometry.

**Fig. 5:**
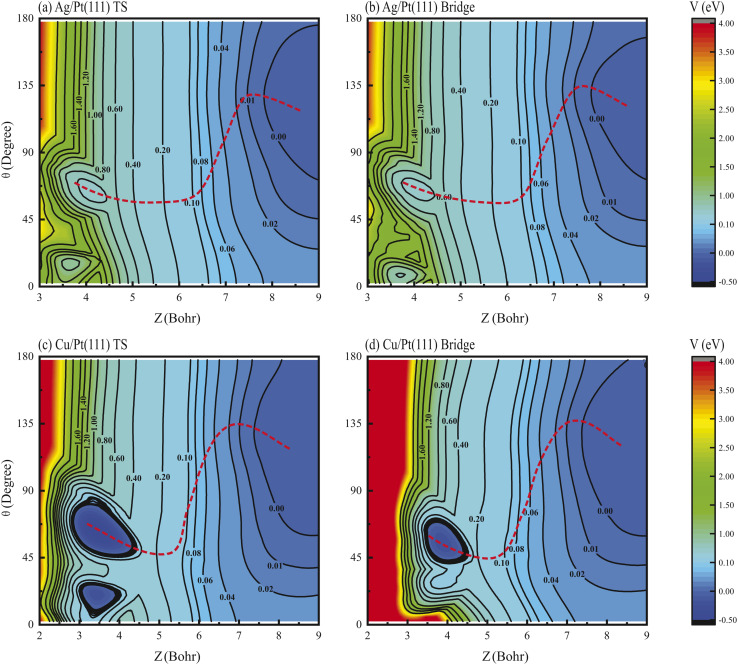
(a) 2D contour plot of the PES for HCl dissociation on the Ag/Pt(111) surface as a function of *Z* and *θ* coordinates with *r* and *ϕ* optimized for the TS site. The contour is relative to the HCl + Ag/Pt(111) asymptote. (b) Same as in (a), except for the bridge site. (c and d) Same as (a and b) except for HCl dissociation on the Cu/Pt(111) surface. The red dashed lines show the MEPs.

For non-rotating HCl (*v* = 0, 1, *j* = 0), the initial polar angle *θ*-distribution follows a Legendre polynomial, exhibiting a maximum centered around *θ* = 90°. The strongly non-monotonic MEP steers the initial wave packet toward angles above 90°, effectively blocking access to the TS region, as demonstrated in detail in prior work for the HCl + Ag/Au(111) reaction.^37^ Consequently, reactivities of non-rotating vibrational states of HCl on Ag/Pt(111) and Cu/Pt(111) surfaces were markedly suppressed, as validated in Fig. 1(a) and (b). Conversely, rotational excitation counteracts this suppression through dual compensatory mechanisms, where the reactivity can be enhanced by redistributing the initial angular probability density or increasing the rotational energy to accelerate molecular reorientation.^39^ These mechanisms collectively rationalize the observed rotational enhancement and alignment inversion phenomena. Supporting evidence comes from rovibrationally excited states (*v* = 1, *j*), where vibrational excitation preserves the initial *θ* distribution while rotational effects dominate the dynamics (Fig. S6 and S7), further confirming the critical role of rotational steering in these reactions.

The trends in rotational efficacy presented in Tables 1 and 2 can be understood within the wave packet steering framework governed by the non-monotonic MEP. Specifically, for a given rotational state *j*, the rotational efficacy *η* is the largest at the highest reaction probability *P*_0_ and decreases as *P*_0_ diminishes. This trend arises from the distinct ways in which *P*(*j* > 0) and *P*(*j* = 0) respond to increasing translational energy. At high energies, which correspond to high *P*_0_, the molecule–surface interaction time is very short. The molecule passes through the anisotropic interaction region too rapidly for the *j* = 0 wave packet to adapt to or overcome the steering effect caused by the non-monotonic MEP, resulting in a deeply suppressed ground-state probability *P*(*j* = 0). In contrast, the reactivity of rotationally excited states (*j* > 0) benefits from their more favorable initial angular distributions and is much less adversely affected by the shortened interaction time. Consequently, the rotational efficacy becomes exceedingly large, as observed at the highest *P*_0_ in the tables. At lower energies where *P*_0_ is smaller, the interaction time is longer. This provides the *j* = 0 wave packet sufficient time to partially overcome the steering effect imposed by the non-monotonic MEP. This greatly narrows the difference between *P*(*j* > 0) and *P*(*j* = 0), thereby drastically lowering the rotational efficacy.

Furthermore, the systematic decrease in rotational efficacy with increasing *j* (observed across each column of the tables) is intricately linked to the changing balance between two competing mechanisms for overcoming the suppression induced by the non-monotonic MEP, as evidenced by the inversion of alignment preference. For low *j* states (*e.g.*, *j* = 1), where rotational energy and speed are modest, the dominant and highly efficient mechanism is the optimal redistribution of the angular wave packet, favoring the cartwheel alignment (*m* = 0). This “steering” strategy leverages the advantageous initial angular distribution to inherently avoid being guided into repulsive regions of the potential. Thereby, it overcomes suppression with minimal energy cost, resulting in a high probability gain per quantum of rotational energy and an overall high efficacy. For high *j* states (*e.g.*, *j* = 8), possessing substantial rotational energy and high speed, the high rotational kinetic energy associated with helicopter alignment (*j* = *m*) enables a “brute-force” traversal against the suppressive steering of the non-monotonic MEP. While this mechanism, competing with and eventually outweighing the steering effect, leads to higher absolute probabilities *P*(*j*), the marginal gain achieved by investing enormous energy into rapid rotation is far less efficient. Consequently, the enhancement efficiency exhibits a diminishing return with increasing *j*. This mechanism is fundamentally distinct from vibrational promotion, which directly adds energy to the reaction coordinate, and explains the differing sensitivity of vibrational and rotational efficacies to translational energy.

The non-monotonic angular dependence of the MEP originates from interfacial charge transfer, as previously established for HCl dissociation on Ag/Au(111) and Cu/Au(111) systems.^37,41^ Bader charge analysis^50,51^ was performed on the Pt(111), Ag/Pt(111) and Cu/Pt(111) surfaces for HCl adsorbed at the top sites in the asymptotic region (*Z* = 7.56 bohr, *r* = 2.46 bohr) and is shown in Table 3. Consistent with HCl's heteronuclear character, charge transfer from H to Cl was observed across all systems. The first-layer Pt atoms on pure Pt(111) gained a charge of 0.0407*e*^−^, indicating electronegativity comparable to pure Ag(111) and Cu(111). In contrast, the Ag and Cu monolayers on Ag/Pt(111) and Cu/Pt(111) lost charges of 0.0918*e*^−^ and 0.1788*e*^−^, respectively, confirming electropositivity, consistent with the results on the Ag/Au(111) and Cu/Au(111) surfaces.^37,41^ This charge depletion primarily arises from the lower work functions of Ag and Cu relative to Pt, driving electron transfer from Ag/Cu monolayers to the Pt substrate. The resulting electrostatic interactions critically steer molecular orientation, where electropositive surface Ag/Cu atoms attract the electronegative Cl atom, stabilizing high-*θ* (120–150°) configurations in the asymptotic region, consistent with the PES topographies shown in Fig. 5.

**Table 3:** Bader charge gain or loss for the H, Cl and atoms of the top first layer for pure Pt(111), Ag/Pt(111) and Cu/Pt(111) surfaces in the asymptotic region, where the HCl molecule is 7.56 bohr above the surface at the top site, with the bond length *r* equaling 2.46 bohr. The positive and negative values refer to the gain and loss of charge (in units of *e*^−^), respectively

	Pt(111)	Ag/Pt(111)	Cu/Pt(111)
H atom	−0.3632	−0.3632	−0.3645
Cl atom	0.3682	0.3757	0.3777
1st layer	0.0391	−0.0918	−0.1788

Transitioning to the interaction region, Bader charge analysis reveals analogous net charge trends, though first-layer Pt atoms on Pt(111) exhibit charge loss and electropositivity. Critically, strong Pauli repulsion between surface atoms (Ag/Cu/Pt) and the electron-dense Cl atom dominates, overwhelming residual electrostatic attraction. This repulsion forces Cl away from the surface, promoting H-down configurations. This compels a directional shift to low-*θ* (40–60°) geometries, clearly visualized in the PES topographies in Fig. 5, particularly at the TS. The resulting H-down orientation at the TS aligns with established geometries for HCl dissociation on diverse metal surfaces^24,26,27,29,35,37,40,41^ and other heteronuclear molecules including H_2_O and CH_4_ dissociation reactions.^17,19,20,31,32,36^ Therefore, the dual-regime interplay of long-range electrostatic attraction and short-range Pauli repulsion between the Cl atom and metal atom generates the characteristic non-monotonic *θ*-dependence on the MEP.

Integrating findings from our prior investigations of HCl dissociation on Ag/Au(111) and Cu/Au(111) surfaces,^39,41^ we establish a robust design principle where maximal rotational enhancement in bimetallic systems is achieved when the substrate work function (*Φ*_sub_) substantially exceeds that of the supported metal (*Φ*_sup_). The critical work function difference (Δ*Φ* = *Φ*_sub_ − *Φ*_sup_ > 0) induces electron transfer from the supported monolayer to the substrate, creating electropositive surfaces that generate strong non-monotonic *θ*-dependence in the MEP through interfacial charge transfer. This topography drives three hallmark dynamical consequences: suppressed reactivity for non-rotating states (*j* = 0), enhanced efficacy for rotationally excited states (*j* > 0), and *j*-dependent inversion of alignment preferences characterized by initial cartwheel dominance (molecular rotation axis perpendicular to the surface), as consistently demonstrated in the title systems and prior HCl dissociation on Ag/Au(111) and Cu/Au(111) studies.^39,41^

To elevate the proposed design principle to a quantitative and predictive level, we establish a semi-quantitative scaling relationship based on data consolidated from the four relevant systems for HCl dissociation on Ag/Au(111), Cu/Au(111), Cu/Pt(111), and Ag/Pt(111) bimetallic alloy surfaces.^39,41^ The work-function differences (Δ*Φ*) for these systems are derived from established literature values.^52^Table 4 summarizes the key parameters for the four model systems, including Δ*Φ*, the net Bader charge change of the supported metal monolayer (Δ*Q*), rotational efficacy (*η*) at a fixed probability (*P*_0_ = 0.01) for HCl initially in the (*v* = 0, *j* = 1) state, and the surface strain state. Fig. 6 plots *η* as a function of Δ*Φ*, with the strain state annotated for each data point.

**Table 4:** The Δ*Φ*(*Φ*_sub_ − *Φ*_sup_), Δ*Q* (the net Bader charge change of the supported metal monolayer), the value *η* of rotational efficacies for HCl initially in the (*v* = 0, *j* = 1) state with dissociation probability *P*_0_ equalling 0.01, and the surface strain state for the four Ag/Au(111), Cu/Au(111), Cu/Pt(111), and Ag/Pt(111) alloy surfaces

Systems	Δ*Φ* (eV)	Δ*Q* (*e*^−^)	*η* (*P*_0_ = 0.01)	Surface strain
Ag/Au(111)	0.57	−0.0609	21.63	0.4% (compressive)
Cu/Au(111)	0.33	−0.1491	2.37	14.44% (tensile)
Cu/Pt(111)	0.72	−0.0918	11.71	8.0% (tensile)
Ag/Pt(111)	0.96	−0.1788	54.57	5.7% (compressive)

**Fig. 6:**
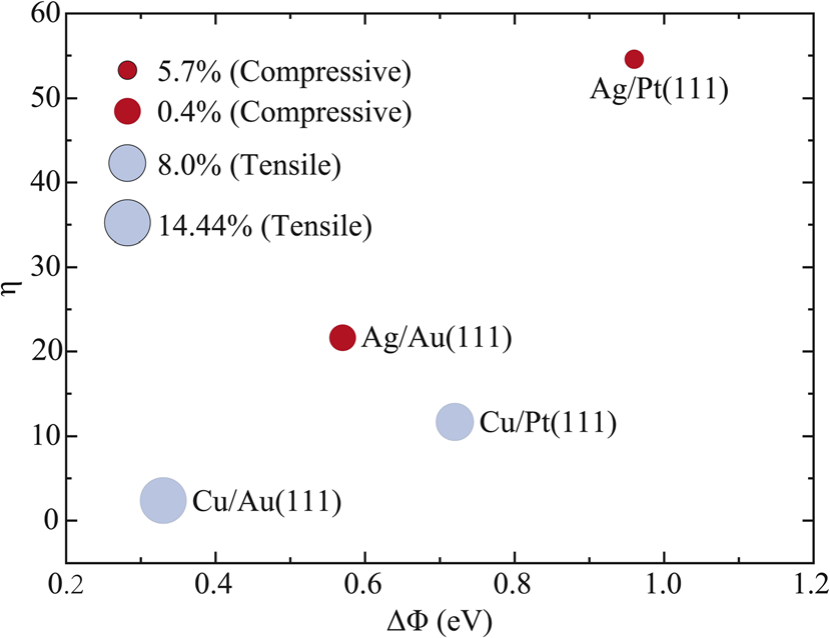
The values *η* of rotational efficacy for HCl initially in the (*v* = 0, *j* = 1) state with dissociation probability *P*_0_ equalling 0.01 as a function of Δ*Φ*(*Φ*_sub_ − *Φ*_sup_) on the four Cu/Au(111), Ag/Au(111), Cu/Pt(111), and Ag/Pt(111) alloy surfaces, with the strain state annotated for each data point.

The data reveal a non-monotonic yet interpretable relationship, offering deeper mechanistic insight than a simple correlation. The systems with the most extreme Δ*Φ* values, Ag/Pt(111) (largest) and Cu/Au(111) (smallest), correspond to the highest and lowest *η* values, respectively, confirming Δ*Φ* as a crucial governing parameter. However, the relationship exhibits a rich structure and underscores a critical, counterintuitive role for surface strain. Tensile strain is well known to enhance absolute reactivity by lowering barriers; our data demonstrate that it systematically suppresses the rotational efficacy *η*. In contrast, compressive strain, which typically inhibits absolute reactivity, promotes a higher *η*. This opposing influence of strain on *η* offers a consistent framework to explain the key observations. First, Ag/Au(111) with a Δ*Φ* of 0.57 eV and 0.4% compression exhibits a higher *η* than Cu/Pt(111), which has a larger Δ*Φ* of 0.72 eV but 8.0% tension. This is because the compressive strain in Ag/Au(111) favors high relative enhancement, whereas the tensile strain in Cu/Pt(111) counteracts its greater electronic driving force. Second, the two extreme cases reinforce this interpretation. Cu/Au(111) combines the smallest Δ*Φ* with substantial tension (14.44%) resulting in the lowest *η*. Conversely, Ag/Pt(111) combines the largest Δ*Φ* with moderate compression (5.7%), yielding the highest *η*.

Furthermore, the chemical identity of the supported metal may provide secondary modulation, as suggested by differences not fully explained by strain alone. This is most clearly reflected in the non-parallel trends observed between Δ*Φ* and the calculated Δ*Q*. Specifically, Cu/Au(111) possesses the smallest Δ*Φ* yet exhibits the second largest Δ*Q*, surpassed only by Ag/Pt(111). This indicates that element-specific interfacial bonding characteristics supplement the charge transfer primarily driven by the work-function difference.

Therefore, we conclude that the rotational efficacy is governed by a clear hierarchy of influencing factors. It is primarily scaled by the work-function difference Δ*Φ*, which establishes the foundational electronic potential for rotational steering. This primary relationship is subsequently modulated in opposite directions by surface strain, where compressive strain promotes *η* while tensile strain suppresses it. Finally, the specific chemical properties of the metals involved provide an additional layer of fine-tuning. This integrated understanding leads to a predictive framework of the form *η* ≈ *f*(Δ*Φ*) × *g*(strain, metal). This formulation transforms the initial qualitative principle into a multivariate design tool, explicitly accounting for the concerted effects of the electronic structure, surface geometry, and chemical identity in governing rotational control.

Conversely, monometallic surfaces and reversed work function gradient bimetallics (*e.g.*, Au/Ag(111)) for HCl dissociation reactions exhibit fundamentally distinct behavior.^24,26,27,29,37,40,41,48^ Due to the absence of unidirectional charge transfer toward substrate-induced electropositivity, first-layer atoms typically exhibit electronegativity in the asymptotic region (*e.g.*, Pt in Pt(111): −0.0407*e*^−^), mediating attractive interactions with the H atom. This contrasts sharply with the electropositive monolayers generated in Δ*Φ* > 0 systems, leading to monotonic *θ*-dependence of the MEP. This mechanistic framework extends to polyatomic systems including H_2_O and CH_4_ dissociation on monometallics.^5,31,32^ Notably, H_2_ dissociation constitutes a special case where charge transfer plays a negligible role. Due to its characteristic TS geometry with the H–H bond essentially parallel to the surface at 90°, H_2_ dissociation systems exhibit rigidly monotonic *θ*-dependence along 90° on all surfaces including monometallic, bimetallic, and single-atom alloys.^35,39,53^ In such linear MEP systems, rotational dynamics are universally characterized by persistent helicopter alignment preference (rotation axis parallel to the surface), unimpaired reactivity for vibrational ground states (*v* = 0, *j* = 0) due to near-identical *θ*-distributions to maximally reactive *j* = *m* states, and less effective promotion of dissociation by rotational excitation than by equivalent translational energy. This paradigm explains rotational suppression observed in prototypical systems including H_2_ + Cu(111), HCl + Ag(111), D_2_O + Ni(111), and CH_4_ + Ni(100).^1,2,5,14,26,30–32^

Notably, HCl + Au/Cu(111) constitutes a special case where rotational enhancement occurs despite monotonic *θ*-dependence, attributed to uniformly tight saddle points constraining all impact sites, highlighting that global PES constraints beyond angular dependence govern rotational control.^40^ Collectively, these observations establish that rotational effects depend predominantly on the global PES topography, diverging fundamentally from vibrational promotion mechanisms governed by TS geometry per Polanyi's rules and the SVP model.

The present analysis, which establishes the global PES topography as the governing factor for rotational steering, is based on quantum dynamics calculations performed on a frozen surface. A natural consideration is how the inclusion of surface atom motion might modify the effective potential energy landscape and thereby influence the predicted effects. Surface atom motion would cause local, temporal displacements of atoms from their equilibrium positions, effectively smearing out the fine details of the static PES topography along the orientational coordinate. For the rotational steering mechanism reported here, which relies on a stark, static non-monotonic angular dependence, the primary effect of this dynamical disorder would likely be a quantitative reduction in the absolute magnitude of the rotational efficacy (*η*), and a broadening of the reaction probability curves, as the contrast between steering-favorable and unfavorable orientations is diminished.

However, two key arguments support the qualitative robustness of our conclusions. First, for the closely related HCl + Au(111) system, an explicit study incorporating surface atom motion and electronically non-adiabatic effects concluded their influence is only modest.^29^ Second, and more fundamentally, the direction of the effect predicted by our static model would remain unchanged. Surface atom fluctuations are not expected to reverse the work-function-driven charge transfer nor invert the created non-monotonic angular dependence, as they would likely only attenuate its amplitude. Therefore, while a more complete model including surface atom motion would provide quantitative refinements, it is not anticipated to qualitatively alter the hierarchy of rotational efficacy between different systems or invalidate the design principle based on Δ*Φ*. Thus, the frozen-surface model provides a reliable and insightful foundation for the fundamental principles of rotational control established in this work.

To summarize, six dimensional time dependent wave packet (TDWP) calculations were conducted for HCl dissociation on Ag/Pt(111) and Cu/Pt(111) bimetallic alloy surfaces, based on two globally accurate PESs. Significant rotational enhancement was observed compared to monometallic systems, with the maximal rotational efficacies reaching roughly 225 and 56 for Ag/Pt(111) and Cu/Pt(111), respectively. This dramatic effect stems from Pt-induced ligand effects, where charge transfer at alloy interfaces generates non-monotonic orientation-dependent potential gradients.

Through systematic investigation of HCl dissociation across multiple bimetallic alloys, we propose a robust design principle, where highly efficient rotational excitation of HCl dissociation on bimetallic alloys is achieved when the substrate work function (*Φ*_sub_) exceeds that of the supported metal (*Φ*_sup_). We further advance this principle by showing that the rotational efficacy *η* is primarily scaled by the work function difference Δ*Φ* and systematically modulated by surface strain, leading to a clear design rule, where *η* is maximized when a large Δ*Φ* is combined with compressive strain. This yields a predictive multivariate framework of the form *η* ≈ *f*(Δ*Φ*) × *g*(strain, metal), which offers a practical strategy for screening and optimizing alloy catalysts without requiring exhaustive dynamical simulations.

The framework resolves long-standing debates about rotational effects in gas-surface reactions by demonstrating their dependence on the global PES topography rather than the TS geometry alone. This provides a unified physical basis that explains not only the curious rotational alignment and efficacy observed in the present HCl alloy systems, but also accounts for the diverse rotational steering phenomena reported earlier for diatomic (H_2_ and HCl) and polyatomic (H_2_O and CH_4_) reactions on pure metal surfaces.^1–3,5,14,18,24,26–32^ It represents a fundamental distinction from the TS localized mechanisms governing vibrational promotion.

Most significantly, our results reveal a broad “quantum state control window”, spanning the range of reaction probabilities where rotational efficacy far exceeds unity, in which molecular rotation acts as a dominant factor governing reactivity. This window is directly defined and accessible through the work function difference descriptor we established. Therefore, the primary practical implication extends beyond enhancing the total rate under thermal equilibrium conditions. Instead, it provides a foundational, tunable principle for designing catalysts where molecular rotation serves as a precise external knob to control reaction pathways or stereoselectivity, advancing the frontier of mode and state selective catalysis.

## Author contributions

T. L.: conceptualization, methodology, software, formal analysis, investigation, writing – original draft, writing – review & editing, supervision, project administration. K. M.: investigation, visualization.

## Conflicts of interest

There are no conflicts to declare.

## Supplementary Material

SC-OLF-D6SC00201C-s001

SC-OLF-D6SC00201C-s002

## Data Availability

All other relevant data are available from the authors upon reasonable request. The data supporting this article have been included as part of the supplementary information (SI). Supplementary information is available. See DOI: https://doi.org/10.1039/d6sc00201c.
