# Supplementary material for: Harnessing work-function-driven rotational steering for quantum state control in HCl dissociation on bimetallic alloys
Source: Chem Sci. 2026 Feb 4;17(12):6187–96. doi: 10.1039/d6sc00201c (PMC12869706; doi:10.1039/d6sc00201c)
Supplement: SC-017-D6SC00201C-s002 [file SC-017-D6SC00201C-s002.zip › support/HCl+AgPtandCuPt_support.pdf]

* To whom corresponding should be addressed: Email: liutianhui@gbu.edu.cn

Supplementary Information

Harnessing Work-Function-Driven Rotational Steering for Quantum State Control in HCl Dissociation on Bimetallic Alloys

Tianhui Liu*[abc], Kaixin Meng[a]

[a]*School of Sciences, Great Bay University, Dongguan 523000, China*

[b]*Great Bay Institute for Advanced Study, Dongguan 523000, China*

[c]*State Key Laboratory of Chemical Reaction Dynamics, Dalian Institute of Chemical Physics, Chinese Academy of Sciences, Dalian, 116023, China*

I. COMPUTATIONAL DETAILS

A. Surface Model and DFT Calculations.

Plane-wave density functional theory (DFT) calculations were conducted using the Vienna *ab initio* Simulation Package (VASP).[1, 2] Electron-ion interactions were described using fully nonlocal optimized projector augmented-wave (PAW) potentials,[3] with Kohn-Sham valence states expanded in a plane-wave basis set.[4] The revised Perdew-Burke-Ernzerhof (RPBE) functional[5] within the generalized gradient approximation (GGA)[6] was employed for exchange-correlation effects. Computational parameters were maintained identical to prior studies of HCl dissociation on Ag/Au(111) and Cu/Au(111) surfaces[7, 8] to ensure consistency.

Alloy surfaces were modeled using a four-layer slab with a 2×2 surface unit cell (1/4-ML coverage). A 16 Å vacuum region was implemented between two repeated cells. Electronic structure calculations utilized a 6×6×1 Monkhorst-Pack k-points grid[9] and a 400 eV plane-wave kinetic energy cutoff. Optimized bulk lattice constants for bulk Ag, Cu and Pt were determined as 4.23 Å, 3.6814 Å and 4.001Å in this work. For bimetallic surfaces, the topmost layer of the Pt(111) slab was replaced with Ag or Cu atoms, resulting in a 5.7% compressive strain for Ag/Pt(111) and 8.0% tensile strain for Cu/Pt(111). Structural optimization was performed for the surface without adsorbates, with the two bottom layers constrained. These fixed coordinates were retained during subsequent reaction calculations to represent a rigid surface.

The HCl dissociation coordinate on metal surfaces are defined in Fig. S1 (a), where the x and y axes are parallel to the surface, Z is the perpendicular distance from the HCl center of mass (COM) to the surface, r is the internuclear distance of HCl, θ is the polar angle and ϕ is the azimuthal angle.

B. Potential Energy Surface

The climbing-image nudged elastic band (CI-NEB) method[10, 11] was employed to determine the transition states (TSs) and minimum energy paths (MEPs) for the dissociative chemisorption of HCl on Ag/Pt(111) and Cu/Pt(111) surfaces, respectively. Each MEP was discretized by five images between the initial state (IS) and final state (FS). Energies

and geometries of the three stationary points (IS, TS, FS) along the MEP are displayed in Fig. S2, with optimized TS geometries provided in Table S1. For HCl+Ag/Pt(111), the physisorbed HCl was assigned as the IS, exhibiting an energy of -0.004 eV relative to the reactant HCl+Ag/Pt(111) asymptote. At this state, the COM of HCl was positioned 9.33 bohr above the top site. The TS was located at 4.53 bohr above the surface with the HCl bond length r stretched to 3.58 bohr, having an energy of 0.95 eV. The FS corresponded to co-adsorbed H and Cl atoms at adjacent fcc sites with an energy of 0.41 eV. For HCl+Cu/Pt(111), the IS was also identified as a physisorbed HCl at the top site with an energy of -0.005 eV. The TS occured at 4.39 bohr above the surface, exhibiting a stretched bond length r of 3.08 bohr and an energy barrier of 0.30 eV. The FS configuration mirrored that of HCl+Ag/Pt(111) but possessed a lower energy of -0.81 eV.

The full-dimensional potential energy surfaces (PESs) were constructed using feed-forward neural networks (NNs) with two hidden layers, adopting an I-J-K-1 architecture. This framework comprises an input layer with I nodes, two hidden layers containing J and K neurons respectively, and an output layer with a single node corresponding to the potential energy value. To account for the translational periodicity of the surface, ten symmetry functions[12–14] were employed as input descriptors for HCl interactions with both alloy surfaces. Approximately 43,000 and 30,000 DFT energy points were computed for HCl+Ag/Pt(111) and HCl+Cu/Pt(111) systems, respectively. Further details regarding NN methodology and training protocols are provided in referenced works[7, 14, 15].

Convergence properties were rigorously evaluated throughout the fitting procedure. As summarized in Table S2, four distinct fits were obtained with minimal root mean square errors (RMSEs) using varied neural network architectures or identical architectures with different weight/bias initializations for each reaction system. The first fit (denoted NN1 PES) was selected for both systems. Six-dimensional dissociation probabilities were computed using the time-dependent wave packet (TDWP) method on the NN1 PES and three alternative global PESs. Probability curves for both reactions are compared in Figures S3(a)-(b). For HCl+Ag/Pt(111) (Fig. S3(a)), dissociation probabilities exhibit a monotonic increase with kinetic energy. Crucially, results from all four PESs demonstrate quantitative agreement. Identical consistency was also observed for HCl+Cu/Pt(111) (Fig. S3(b)), confirming robust convergence of the NN1 PESs with respect to fitting procedures.

Two dimensional (2D) contour plots of the NN1 PES for the HCl+Ag/Pt(111) reaction

at the fixed bridge and hcp sites as a function of Z and r were illustrated in Fig. S4 (a)-(b), with other coordinates (θ and ϕ) optimized. Corresponding results for HCl+Cu/Pt(111) reaction were shown in Fig. S4 (c)-(d) for comparative analysis. As seen in Fig. S4 (a)-(b) for HCl+Ag/Pt(111), the reaction barriers were located around r = 3.54 bohr and Z = 4.61 bohr, with an energy of 0.94 eV for the bridge site, r = 3.60 bohr and Z = 4.56 bohr, with an energy of 0.98 eV for the hcp site. For HCl+Cu/Pt(111), the reaction barriers were located around r = 3.07 bohr and Z = 4.39 bohr, with an energy of 0.31 eV for the bridge site, r = 3.07 bohr and Z = 4.37 bohr, with an energy of 0.33 eV for hcp site. Collectively, the smooth L-shaped reaction paths (Fig. S4), minimal RMSE values (Table S2), and convergence across NN fitting procedures (Fig. S3) demonstrated rigorous PES fidelity. Consequently, the NN1 PESs were validated as sufficiently accurate for quantum dynamical investigations and adopted as definitive potential energy surfaces for dissociative chemisorption studies.

C. Time Dependent Wave Packet (TDWP) Method

Six-dimensional (6D) time-dependent wave packet (TDWP) methodologies employed in this study follow established protocols from prior work[16–19]. Hence, a concise overview is provided below. The 6D Hamiltonian for the title reaction is expressed in terms of six molecule coordinates (x, y, Z, r, θ , ϕ) (shown in Fig. S1 (a)) as ($\hbar$=1 hereafter),

$$\begin{aligned}\hat{H} = & -\frac{1}{2M}(\frac{1}{sin^2\alpha}\frac{\partial^2}{\partial x^2} - \frac{2cos\alpha}{sin^2\alpha}\frac{\partial^2}{\partial x\partial y} + \frac{1}{sin^2\alpha}\frac{\partial^2}{\partial y^2}) \\ & -\frac{1}{2M}\frac{\partial^2}{\partial Z^2} - \frac{1}{2\mu}\frac{\partial^2}{\partial r^2} + \frac{j^2}{2\mu r^2} \\ & +V(x,y,Z,r,\theta,\phi), \end{aligned} \tag{1}$$

where M, μ and j denote the total mass, reduced mass and rotational angular momentum of HCl, respectively. The last term $V(Z,r,x,y,\theta,\phi)$ is the interaction potential energy. A skewing angle α equaling 120° was adopted for both alloy surfaces.

The time-dependent wave function was expanded in terms of standard Gaussian function $G^0(Z)$, vibrational eigenfunction $\phi_v(r)$, and angular momentum eigenfunctions. The Z and r coordinates were efficiently represented by the sin-DVR (discrete variable representation) basis functions[20, 21]. The angular coordinates (θ and ϕ) were treated using

a direct product of Legendre polynomials $P_n(cos(\theta))$ and $e^{im\phi}$ as good basis representations, implemented by separating the ϕ-dependent term from the $\hat{j}^2$ operator.[22] Wave function propagation was performed using the split-operator method[23], with wavefunction absorption at grid boundaries employed to prevent unphysical reflections[24]. State-resolved dissociation probabilities were obtained by projecting out the energy dependent reactive flux. Additional computational details regarding the TDWP methodology were documented in prior publications[16, 18]. Numerical parameters for 6D quantum dynamics calculations on both alloy surfaces were compiled in Table S3.

---

[1] G. Kresse and J. Furthmüller, *Comp. Mater. Sci.*, 1996, **6**, 15–50.

[2] G. Kresse and J. Furthmüller, *Phys. Rev. B*, 1996, **54**, 11169–11186.

[3] P. E. Blöchl, *Phys. Rev. B*, 1994, **50**, 17953–17979.

[4] G. Kresse and D. Joubert, *Phys. Rev. B*, 1999, **59**, 1758–1775.

[5] B. Hammer, L. B. Hansen and J. K. Nørskov, *Phys. Rev. B*, 1999, **59**, 7413–7421.

[6] J. P. Perdew, K. Burke and M. Ernzerhof, *Phys. Rev. Lett.*, 1996, **77**, 3865–3868.

[7] T. Liu, T. Peng, B. Fu and D. H. Zhang, *J. Phys. Chem. Lett.*, 2023, **14**, 9713–9719.

[8] T. Liu, B. Fu and D. H. Zhang, *Chin. J. Chem. Phys.*, 2025, **38**, 415–426.

[9] H. J. Monkhorst and J. D. Pack, *Phys. Rev. B*, 1976, **13**, 5188–5192.

[10] G. Henkelman, B. P. Uberuaga and H. Jónsson, *J. Chem. Phys.*, 2000, **113**, 9901–9904.

[11] *Nudged elastic band method for finding minimum energy paths of transitions. Classical and Quantum Dynamics in Condensed Phase Simulations*, ed. B. J. Berne, G. Ciccotti and D. F. Coker., World Scientific, Singapore, 1998.

[12] J. Behler, S. Lorenz and K. Reuter, *J. Chem. Phys.*, 2007, **127**, 014705.

[13] B. Jiang and H. Guo, *Phys. Chem. Chem. Phys.*, 2014, **16**, 24704–24715.

[14] H. Shi, T. Liu, Y. Fu, X. Lu, B. Fu and D. H. Zhang, *J. Phys. Chem. C*, 2021, **125**, 23105–23114.

[15] T. Liu, H. Shi, B. Fu and D. H. Zhang, *J. Chem. Phys.*, 2022, **157**, 244702.

[16] T. Liu, B. Fu and D. H. Zhang, *J. Chem. Phys.*, 2014, **140**, 144701.

[17] T. Liu, B. Fu and D. H. Zhang, *J. Chem. Phys.*, 2013, **139**, 184705.

[18] T. Liu, B. Fu and D. H. Zhang, *J. Chem. Phys.*, 2014, **141**, 194302.

[19] T. Liu, B. Fu and D. H. Zhang, *J. Chem. Phys.*, 2017, **146**, 164706.

[20] Z. Bacic and J. C. Light, *Annu. Rev. Phys. Chem.*, 1989, **40**, 469–498.

[21] D. T. Colbert and W. H. Miller, *J. Chem. Phys.*, 1992, **96**, 1982–1991.

[22] J. Dai and J. C. Light, *J. Chem. Phys.*, 1997, **107**, 1676–1679.

[23] J. A. Fleck Jr., J. R. Morris and M. D. Feit, *Appl. Phys.*, 1976, **10**, 129–160.

[24] D. Neuhauser and M. Baer, *J. Chem. Phys.*, 1989, **90**, 4351–4355.

TABLE S1: The transition state (TS) geometries and energies for the dissociative chemisorption of HCl on Ag/Pt(111) and Cu/Pt(111) surfaces, respectively. X, Y are given in units of the surface lattice constant L for the corresponding interacting surface.

surface	X (L)	Y (L)	Z (bohr)	r (bohr)	θ (degree)	ϕ (degree)	E_b (eV)
Ag/Pt(111)	0.325	0.232	4.53	3.58	50.7	13.0	0.95
Cu/Pt(111)	0.509	0.381	4.39	3.08	39.4	344.7	0.30

TABLE S2: Neural network structure parameters and fitting errors (meV) for the four fits with total DFT data points calculated for HCl dissociation on Ag/Pt(111) and Cu/Pt(111) surfaces, respectively.

Reaction systems	HCl+Ag/Pt(111)	HCl+Cu/Pt(111)
Number of data points	43133	29701
Fit1	10-85-75-1 (4.4) (a1)	10-70-60-1 (3.7) (b1)
Fit2	10-85-80-1 (4.8) (a2)	10-80-65-1 (3.9) (b2)
Fit3	10-85-75-1 (5.3) (a3)	10-80-65-1 (3.9) (b3)
Fit4	10-80-75-1 (5.7) (a4)	10-70-65-1 (3.9) (b4)

TABLE S3: The numerical parameters used in the 6D quantum dynamics calculations for the HCl on Ag/Pt(111) and Cu/Pt(111) systems, respectively. Parameter values are in atomic units unless stated otherwise.

Parameter for HCl (v=0, j=0)	Ag/Pt(111)	Cu/Pt(111)
Kinetic energy region	[0.4-2.0] eV	[0.01-1.6] eV
Initial Gauss wave packet	$Z_0 = 14.5, \delta = 0.05, E_0$=1.2 eV	$Z_0 = 14.5, \delta = 0.04, E_0$=0.6 eV
Grid/basis ranges for Z	$Z \in$ [3.0, 17.0]	$Z \in$ [2.0, 17.0]
Grid/basis sizes for Z	N_Z^{asy}=540, N_Z^{int}=160	N_Z^{asy}=550, N_Z^{int}=170
Grid/basis ranges for r	$r \in$ [1.5, 7.0]	$r \in$ [1.5, 7.0]
Grid/basis sizes for r	N_r^{asy}=6, N_r^{int}=45	N_r^{asy}=8, N_r^{int}=50
Grid/basis sizes for angles	j_{max}=45, M_{max}=35	j_{max}=50, M_{max}=40
Grid/basis sizes for x and y	N_x=N_y=11	N_x=N_y=11
Flux surface	r= 5.1	r= 4.9
Time step	Δt= 10	Δt= 10
Total propagation time	27000	36000

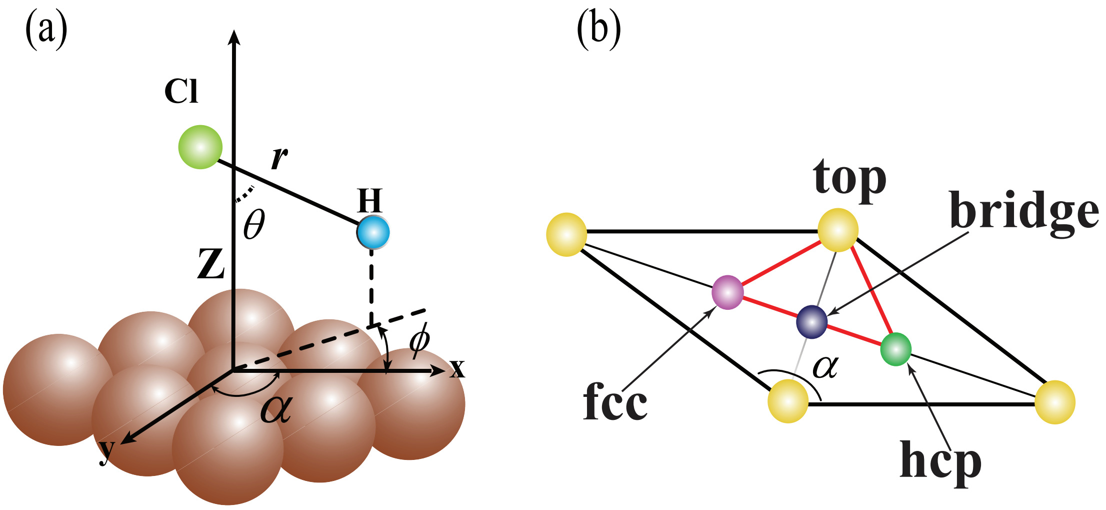

Figure S1: (a) Molecular coordinates for the dissociative chemisorption of HCl on metal surface. (b) The location of the four top, bridge, hcp, and fcc impact sites and the irreducible triangle of Ag/Pt(111) surface unit cell (shown in red triangle).

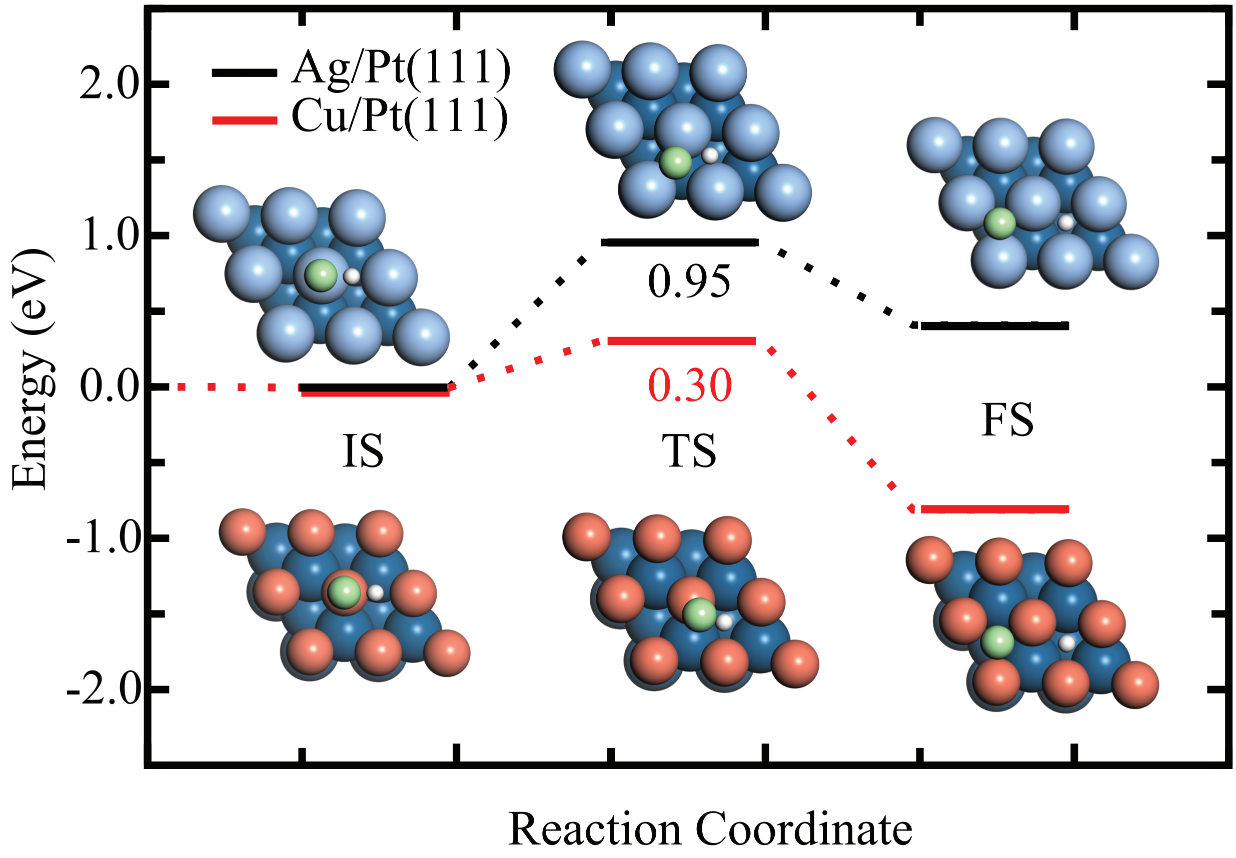

Figure S2: DFT energies and geometries of the stationary points including the physisorption well (IS), transition state (TS) and the product state (FS) obtained by the CI-NEB method for the dissociative chemisorption of HCl on Ag/Pt(111) and Cu/Pt(111) surfaces, respectively.

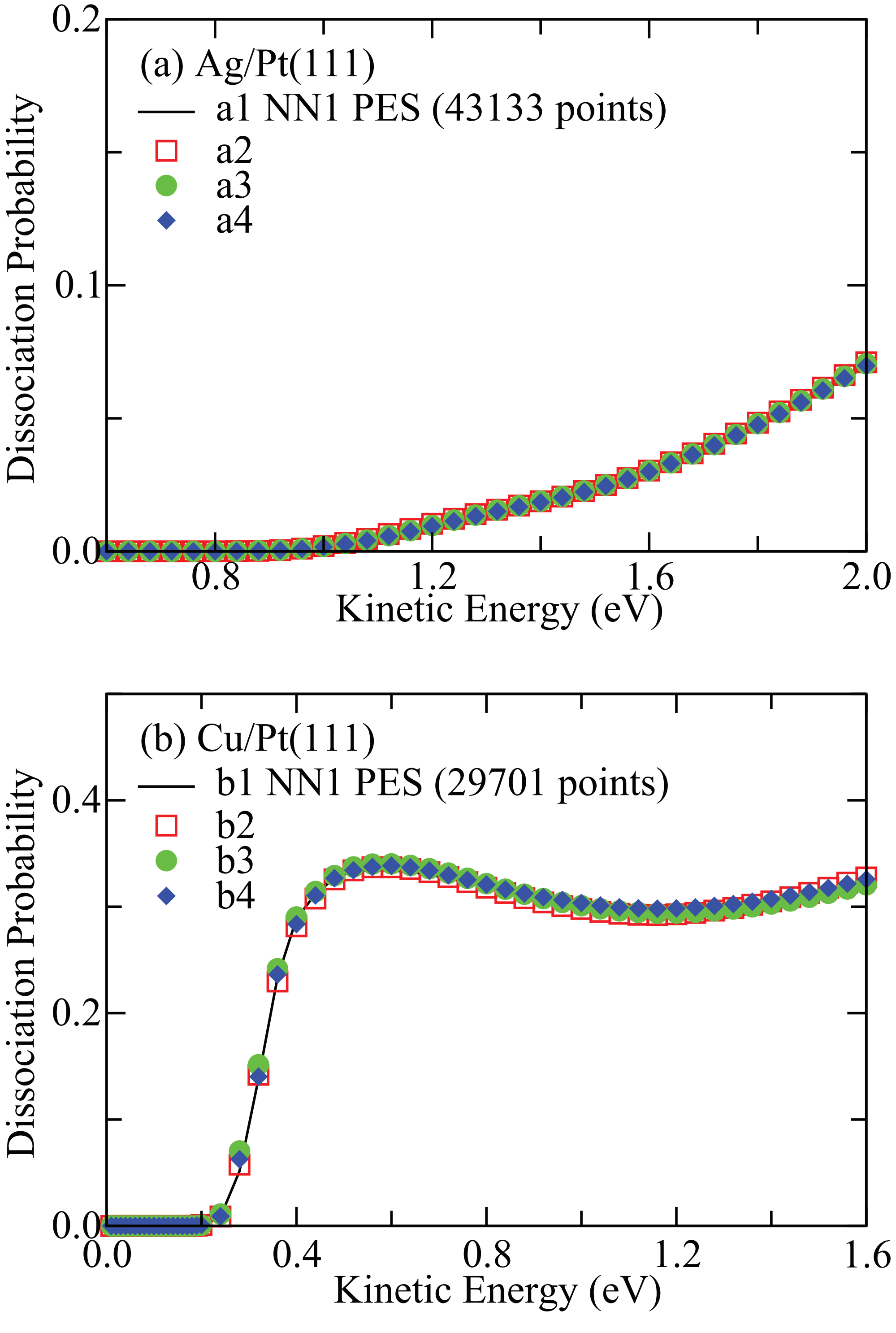

Figure S3: (a) Comparison of the 6D dissociation probabilities for the dissociative chemisorption of HCl (v=0,j=0) on Ag/Pt(111) surface calculated on NN1 and other three PESs. (b) The same as (a) except on the Cu/Pt(111) surface.

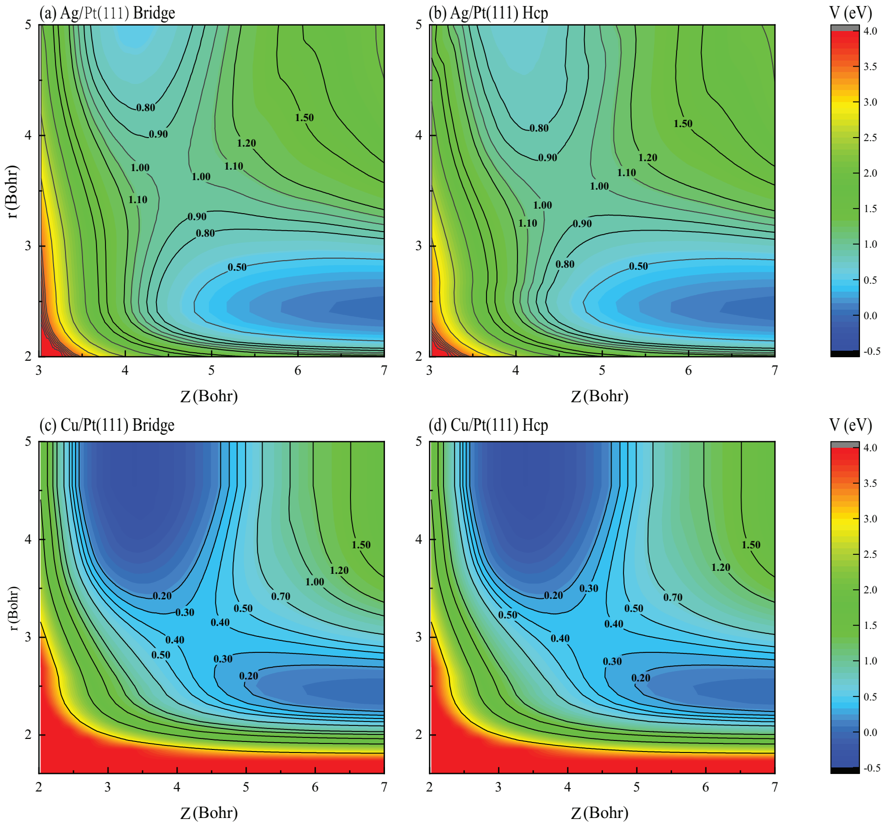

Figure S4: (a) 2D contour plot of the PES as a function of Z and r for the bridge site for HCl on Ag/Pt(111), with other coordinates optimized. (b) Same as (a) except for the hcp site. (c)-(d) Same as (a)-(b) except for HCl dissociation on Cu/Pt(111) surface.

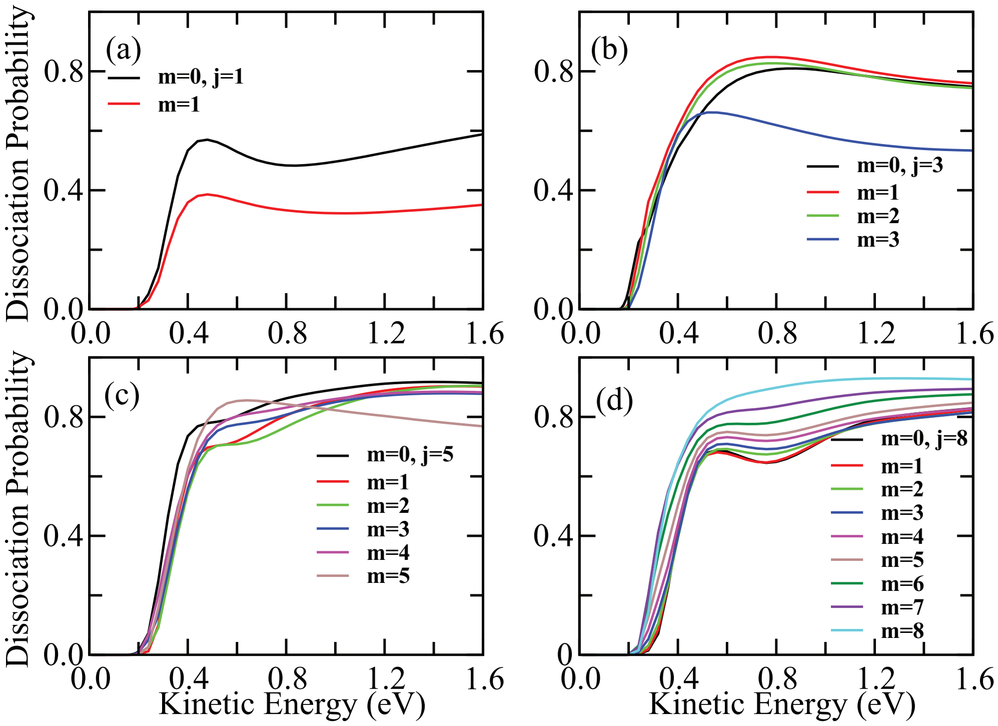

Figure S5: (a) The 6D dissociation probabilities with HCl initially in the same rovibrational state (v=0, j=1) but different alignments (m=0-1) on the Cu/Pt(111) surface. (b), (c), (d) Same as (a) except for HCl initially in (v=0, j=3), (v=0, j=5), and (v=0, j=8) states, respectively.

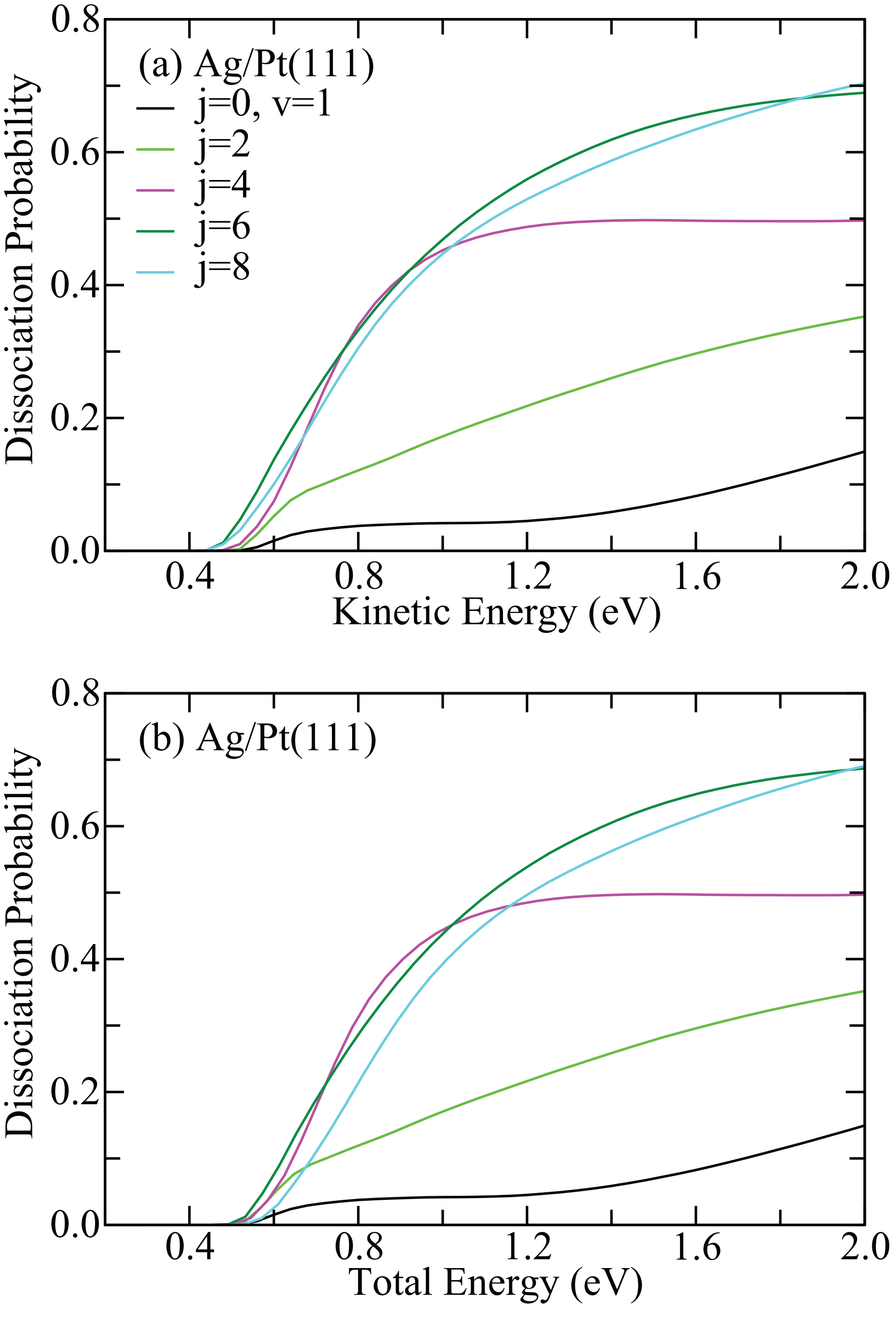

Figure S6: (a) Comparison of the 6D dissociation probabilities as a function of kinetic energy with HCl initially in the rotational states (v=1, j=2, 4, 6, 8) on Ag/Pt(111). (b) The same as in (a), except as a function of total energy.

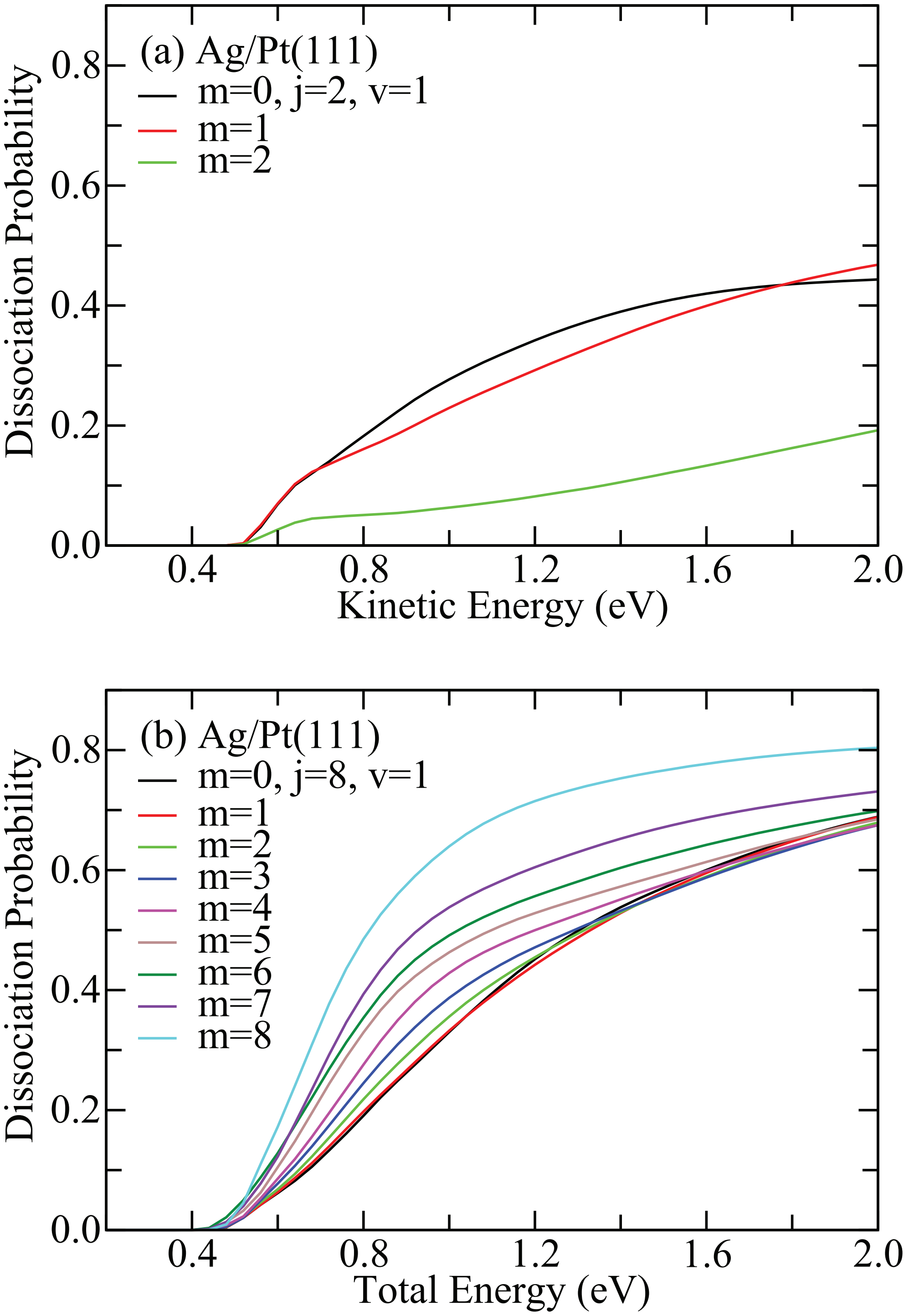

Figure S7: (a) The 6D dissociation probabilities with HCl initially in the same rovibrational state (v=1, j=2) but different alignments (m=0-2) on the Ag/Pt(111) surface. (b) Same as (a) except for HCl initially in (v=1, j=8) state.
