# Supplementary figures and images for: Harnessing work-function-driven rotational steering for quantum state control in HCl dissociation on bimetallic alloys
Source: Chem Sci. 2026 Feb 4;17(12):6187–96. doi: 10.1039/d6sc00201c (PMC12869706; doi:10.1039/d6sc00201c)

(a)

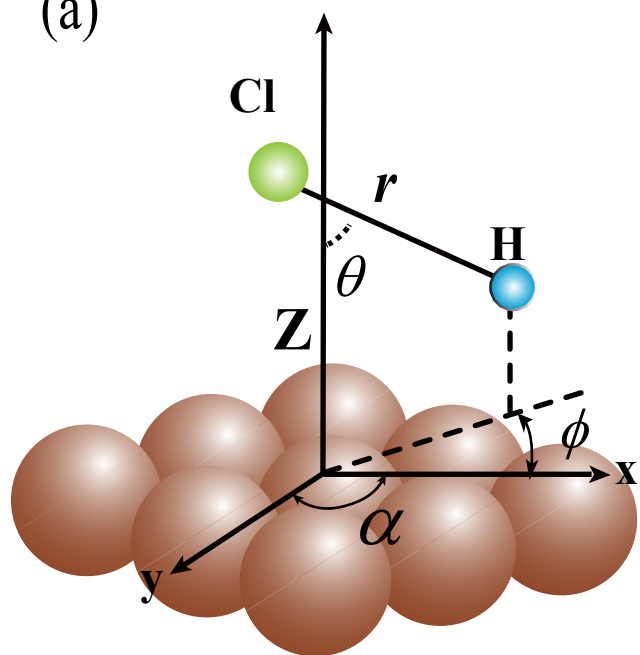

(b)

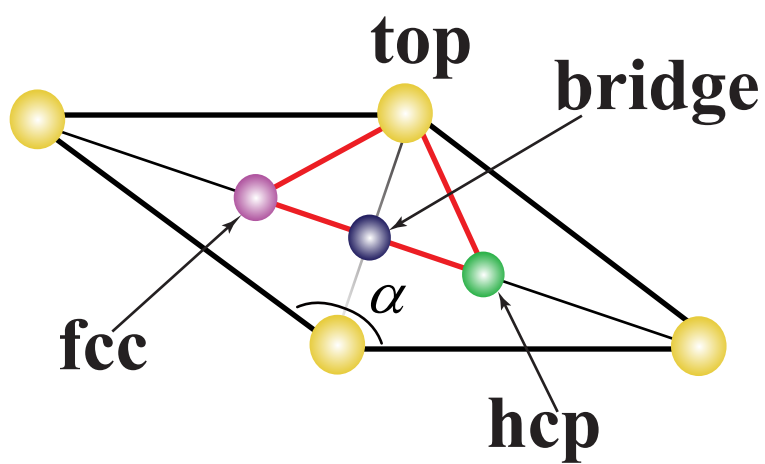

Supplement: SC-017-D6SC00201C-s002 [file SC-017-D6SC00201C-s002.zip › support/FigS1_coordinate.pdf]

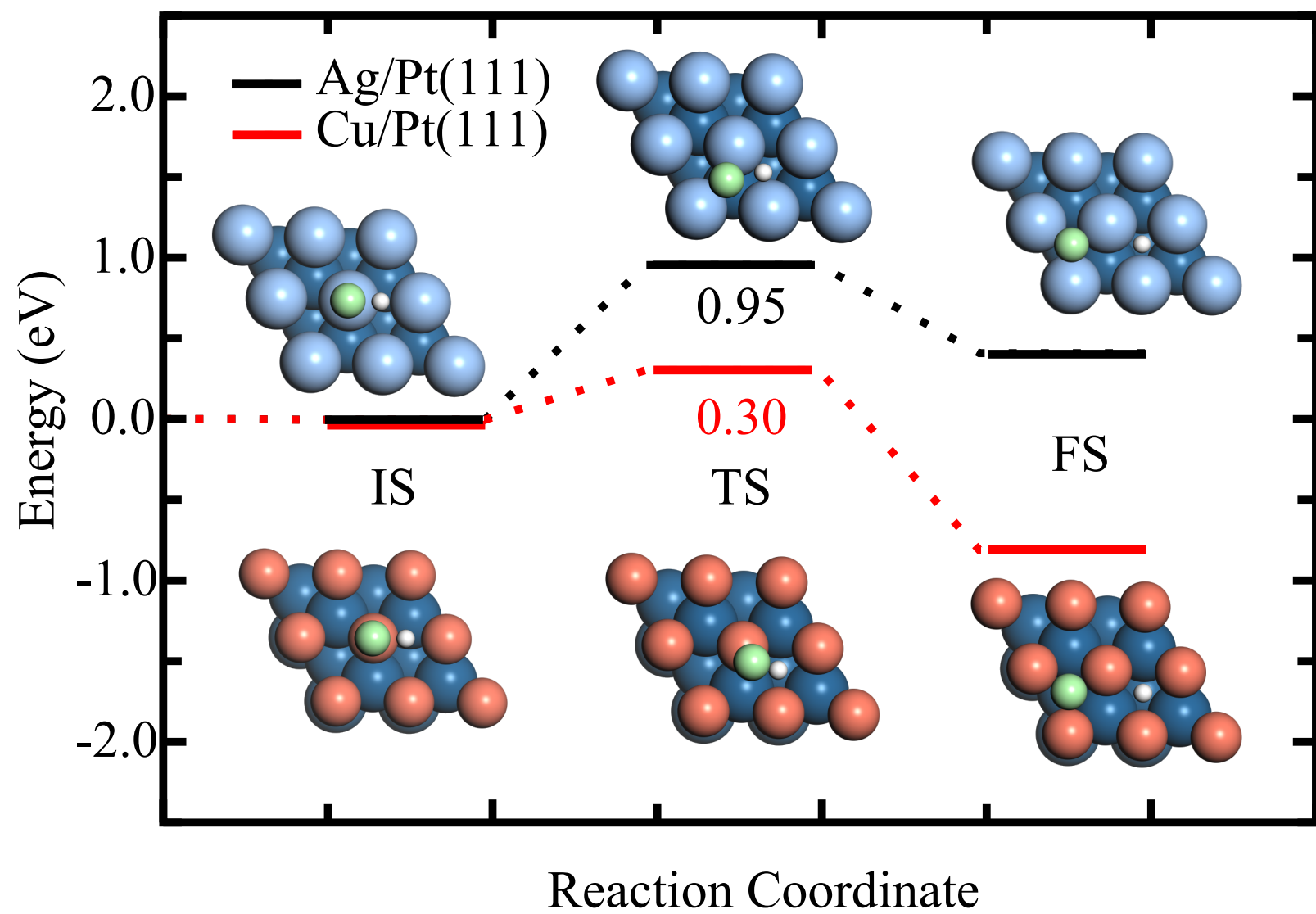

Supplement: SC-017-D6SC00201C-s002 [file SC-017-D6SC00201C-s002.zip › support/FigS2_cineb.pdf]

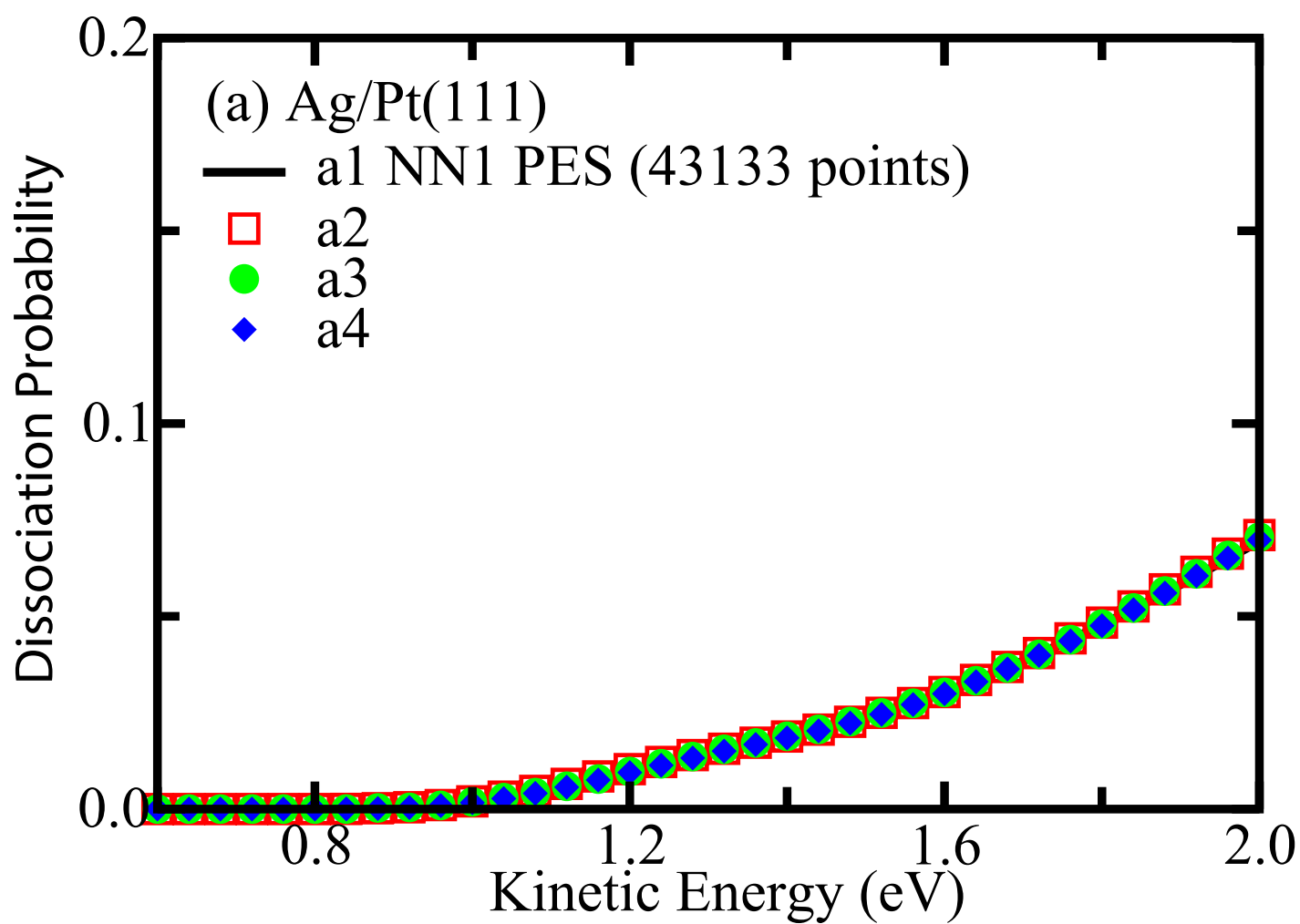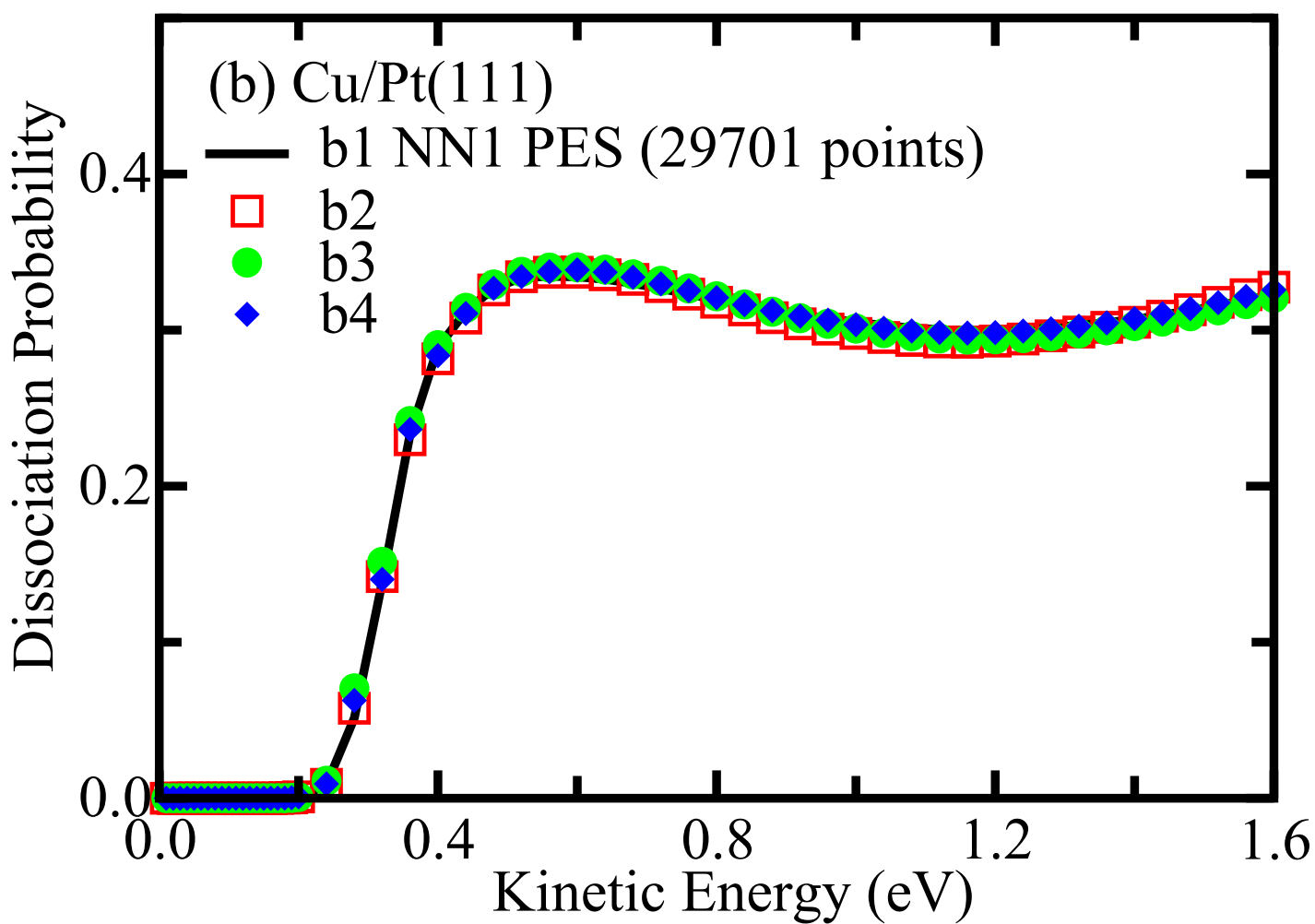

Supplement: SC-017-D6SC00201C-s002 [file SC-017-D6SC00201C-s002.zip › support/FigS3_pes_converg.pdf]

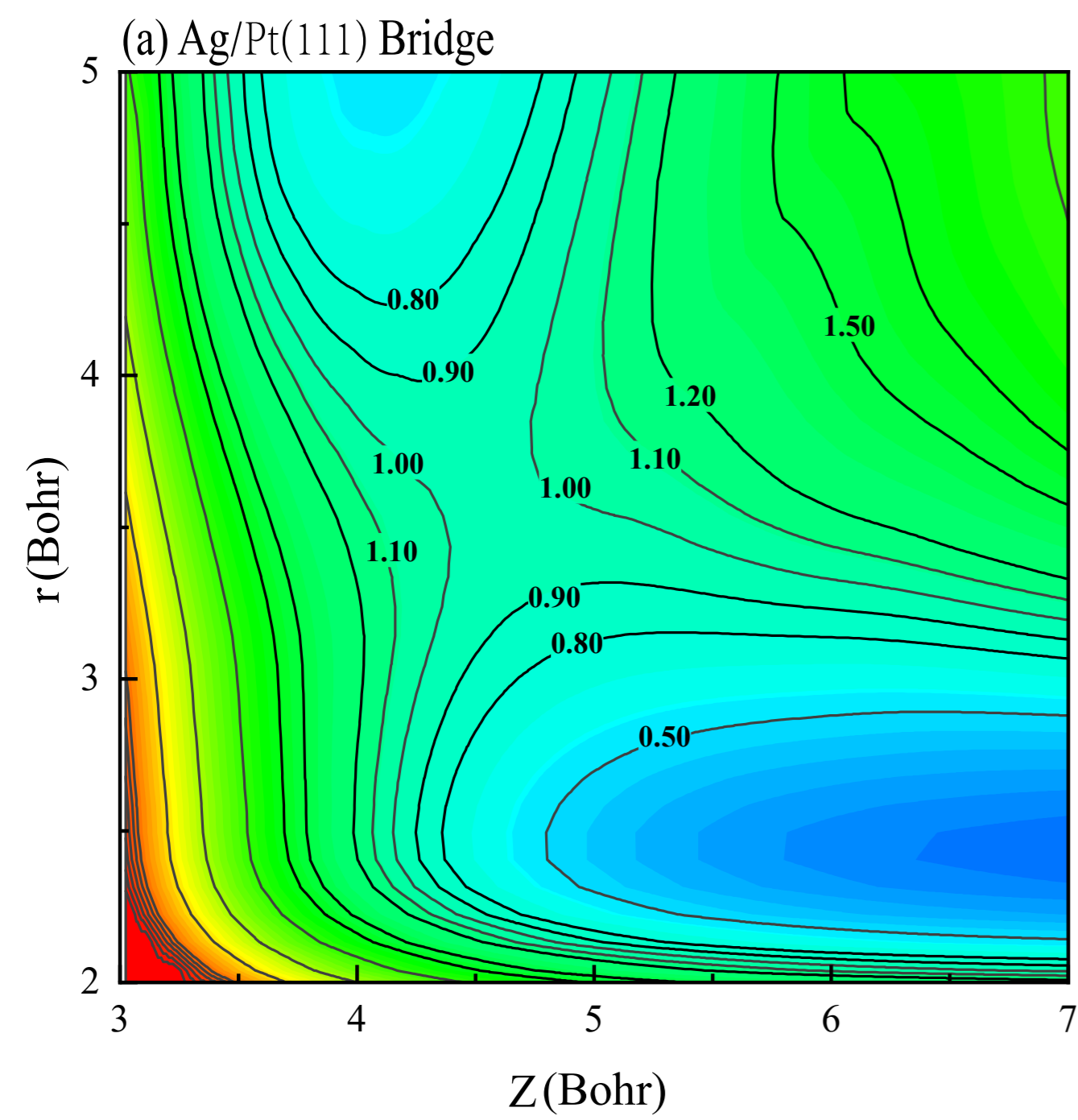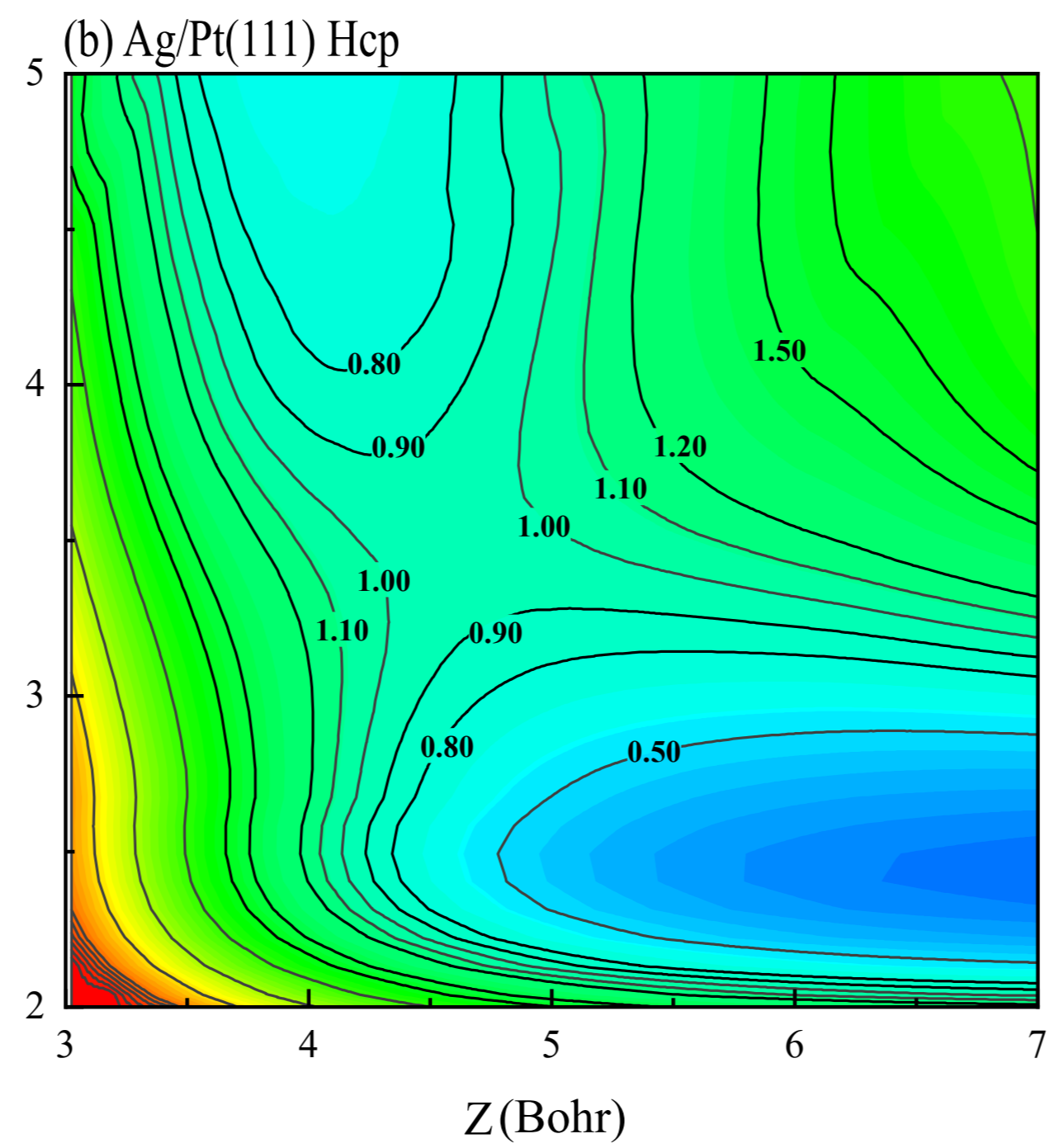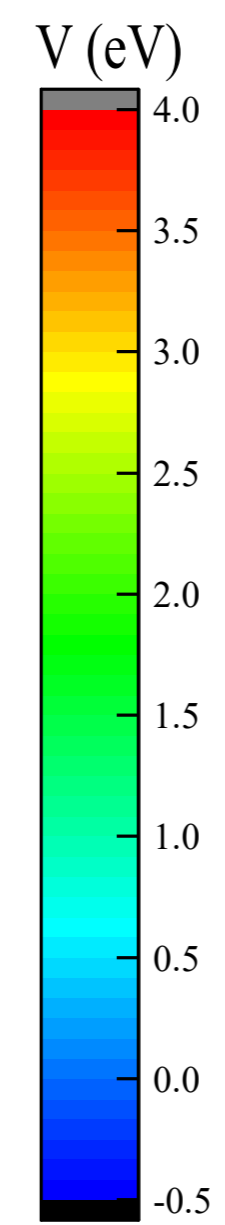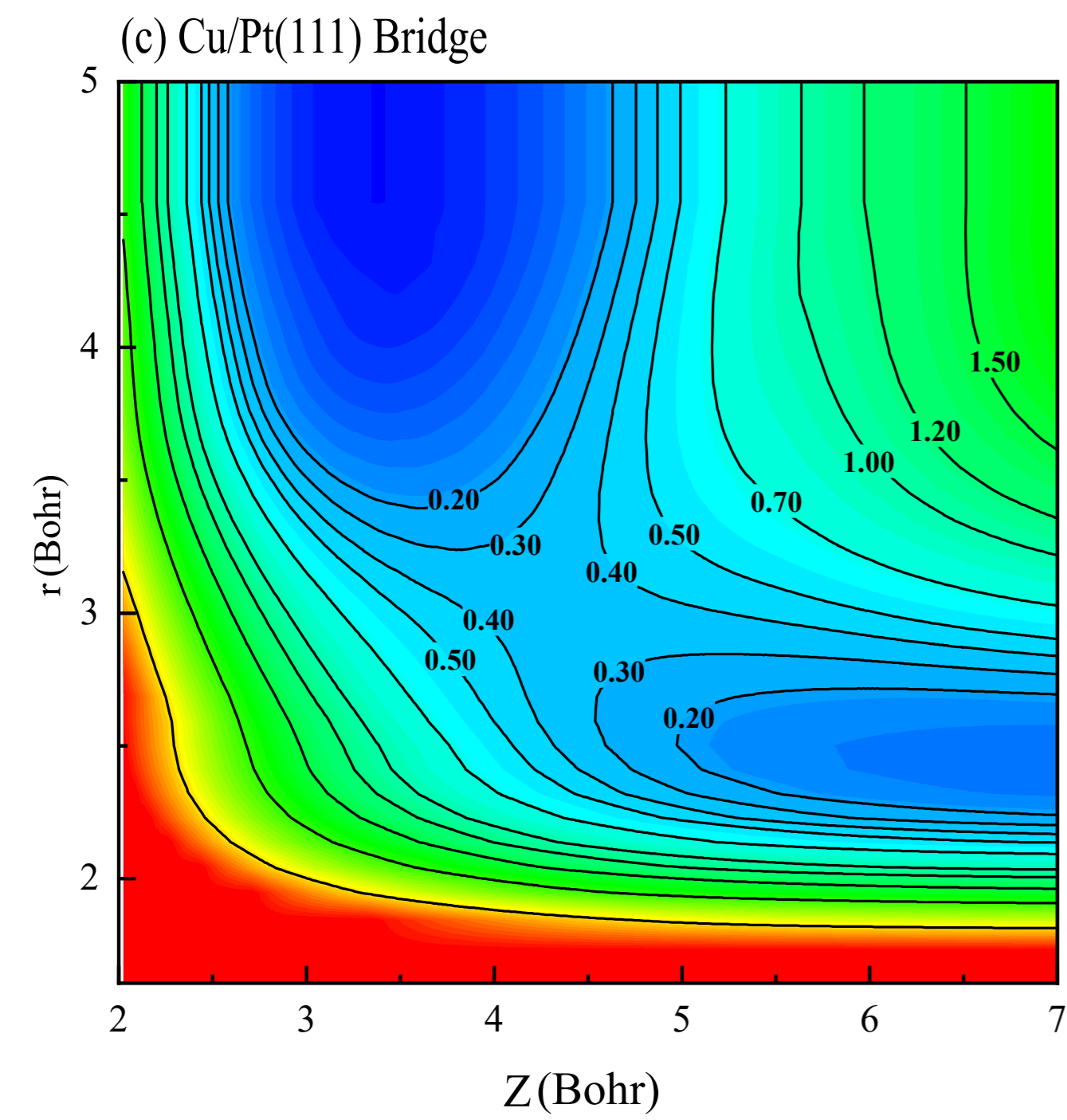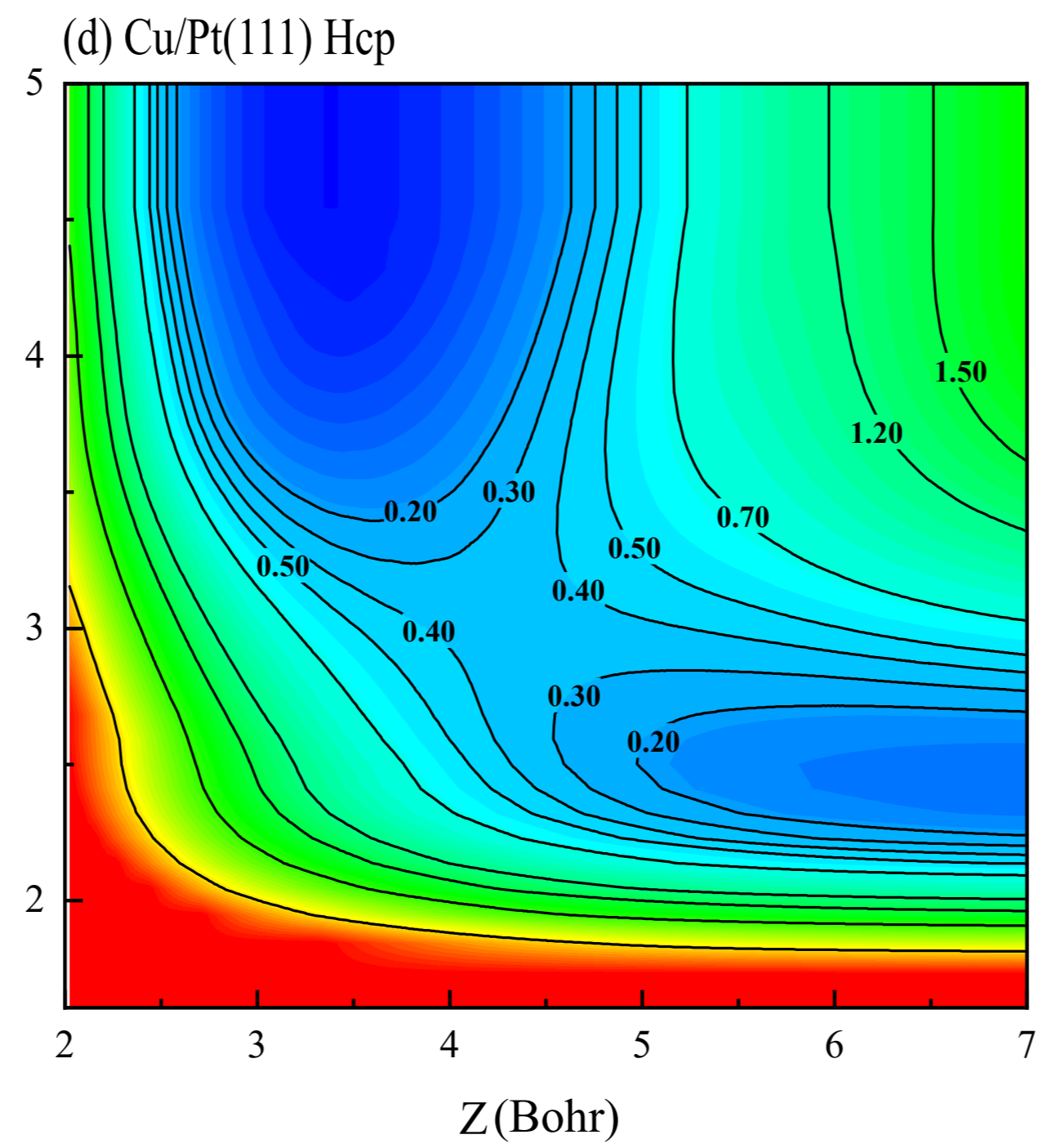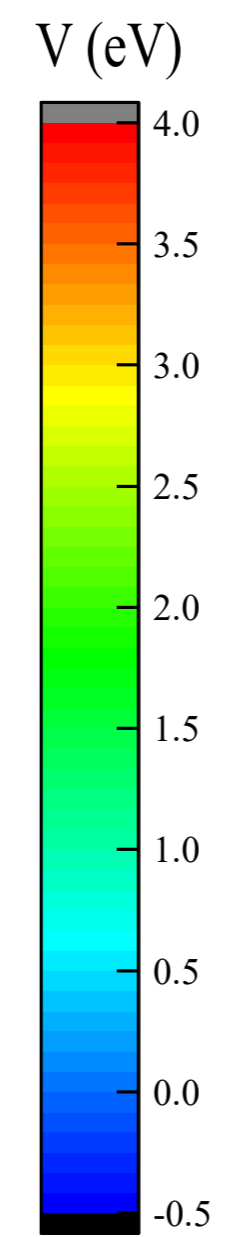

Supplement: SC-017-D6SC00201C-s002 [file SC-017-D6SC00201C-s002.zip › support/FigS4_2D_plot.pdf]

Dissociation Probability

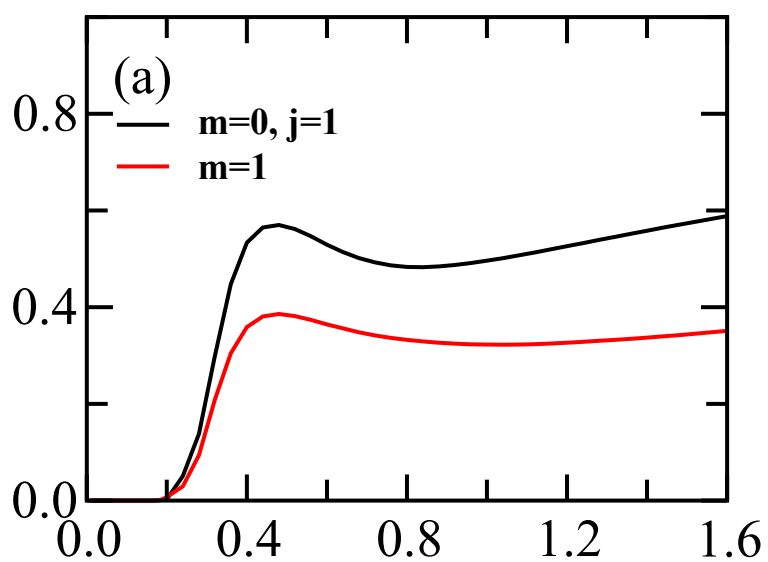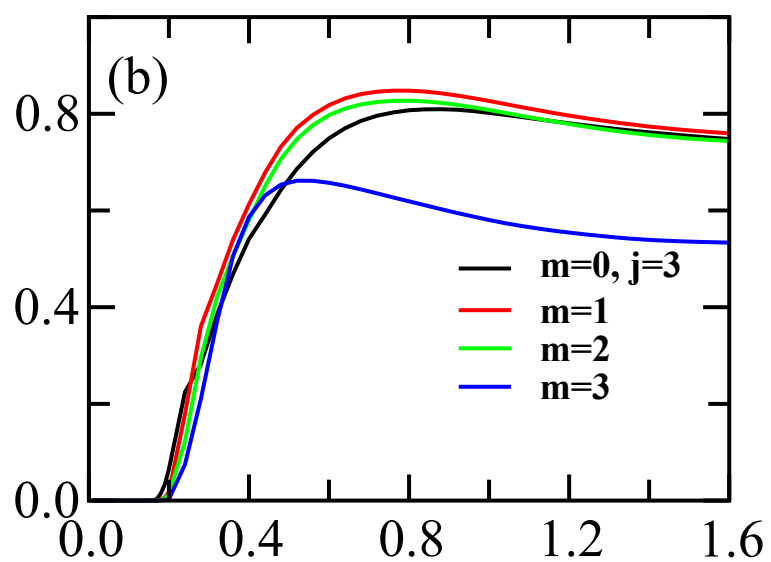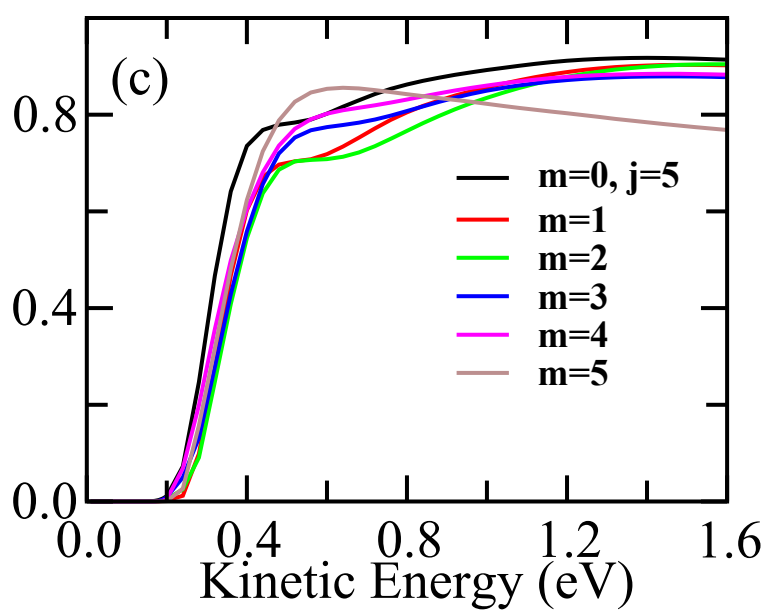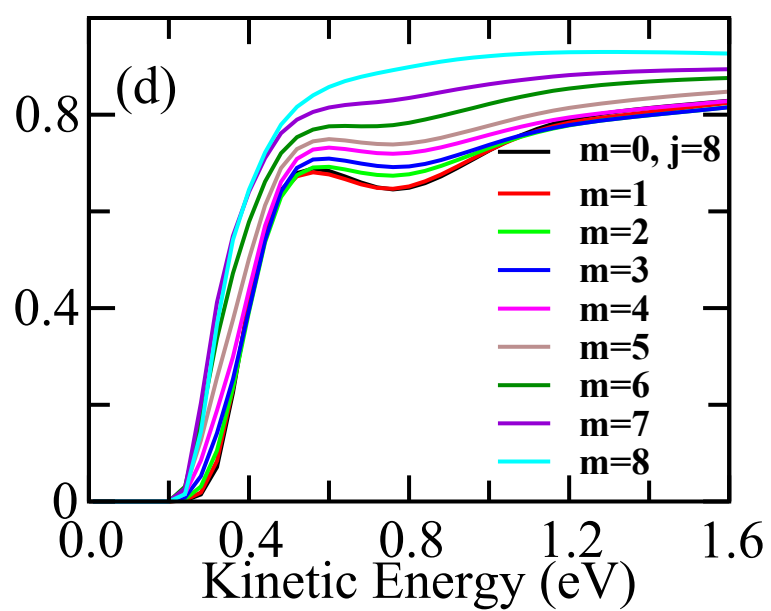

Supplement: SC-017-D6SC00201C-s002 [file SC-017-D6SC00201C-s002.zip › support/FigS5_oritation_j_CuPt.pdf]

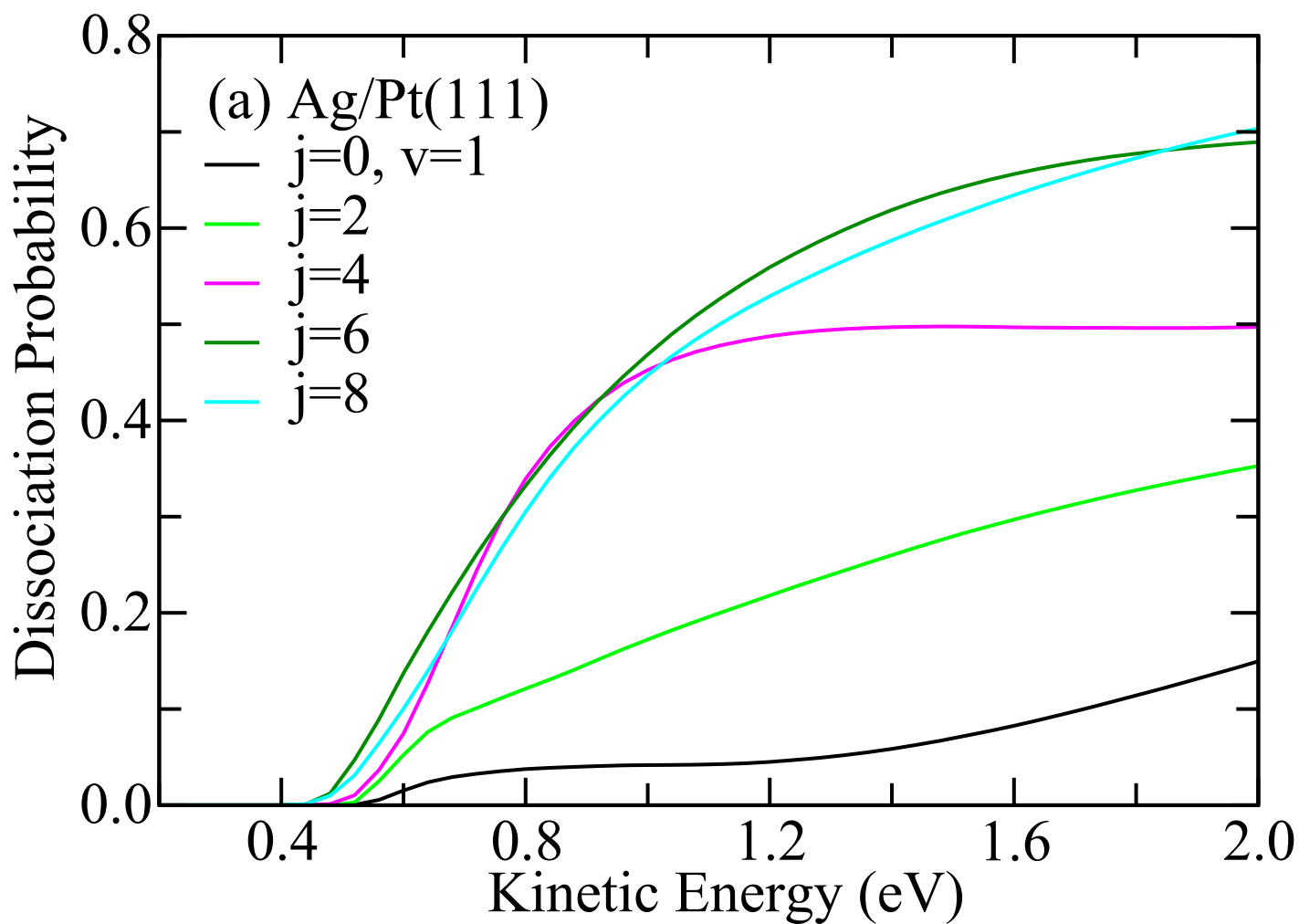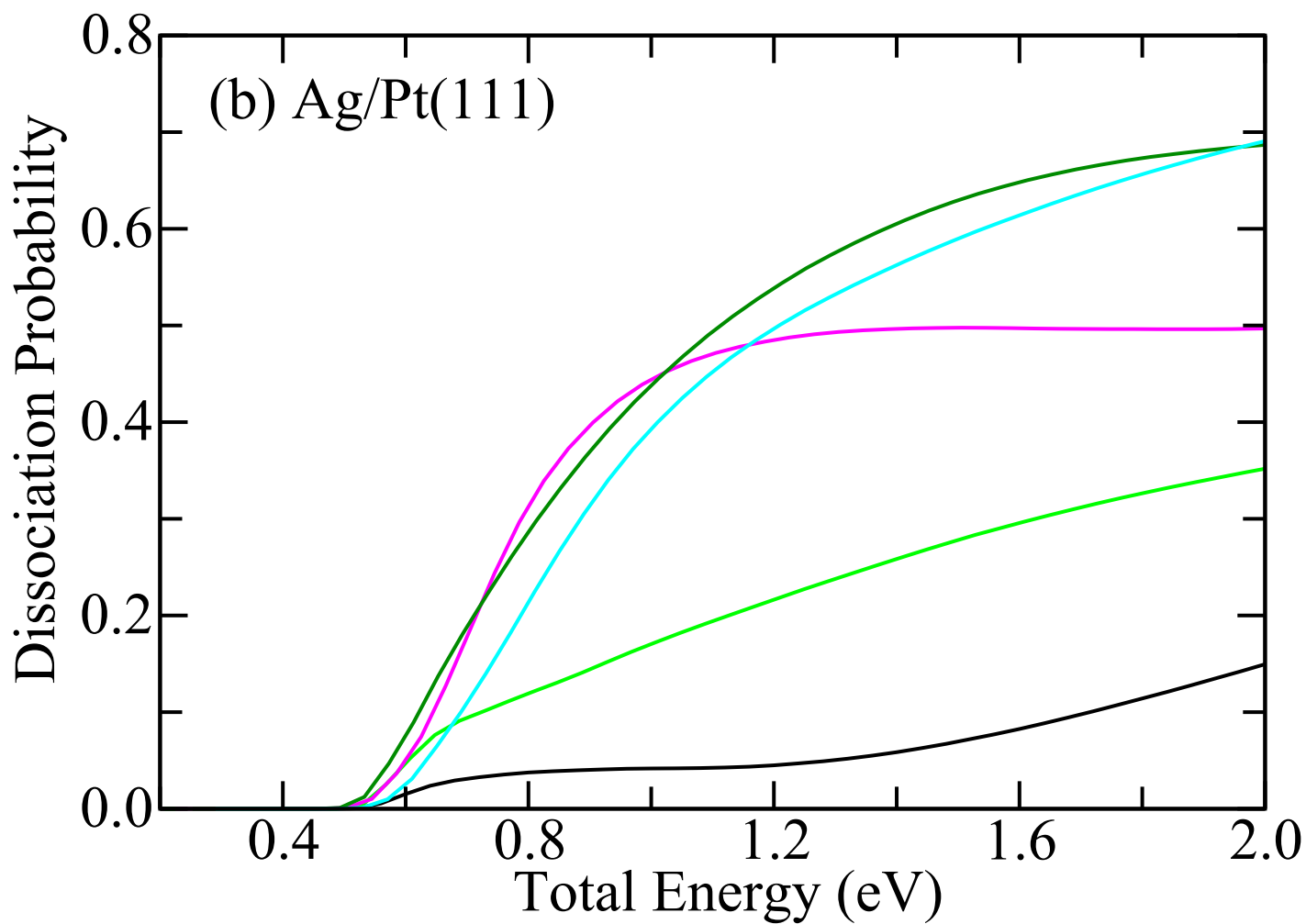

Supplement: SC-017-D6SC00201C-s002 [file SC-017-D6SC00201C-s002.zip › support/FigS6_AgPt_v1j.pdf]

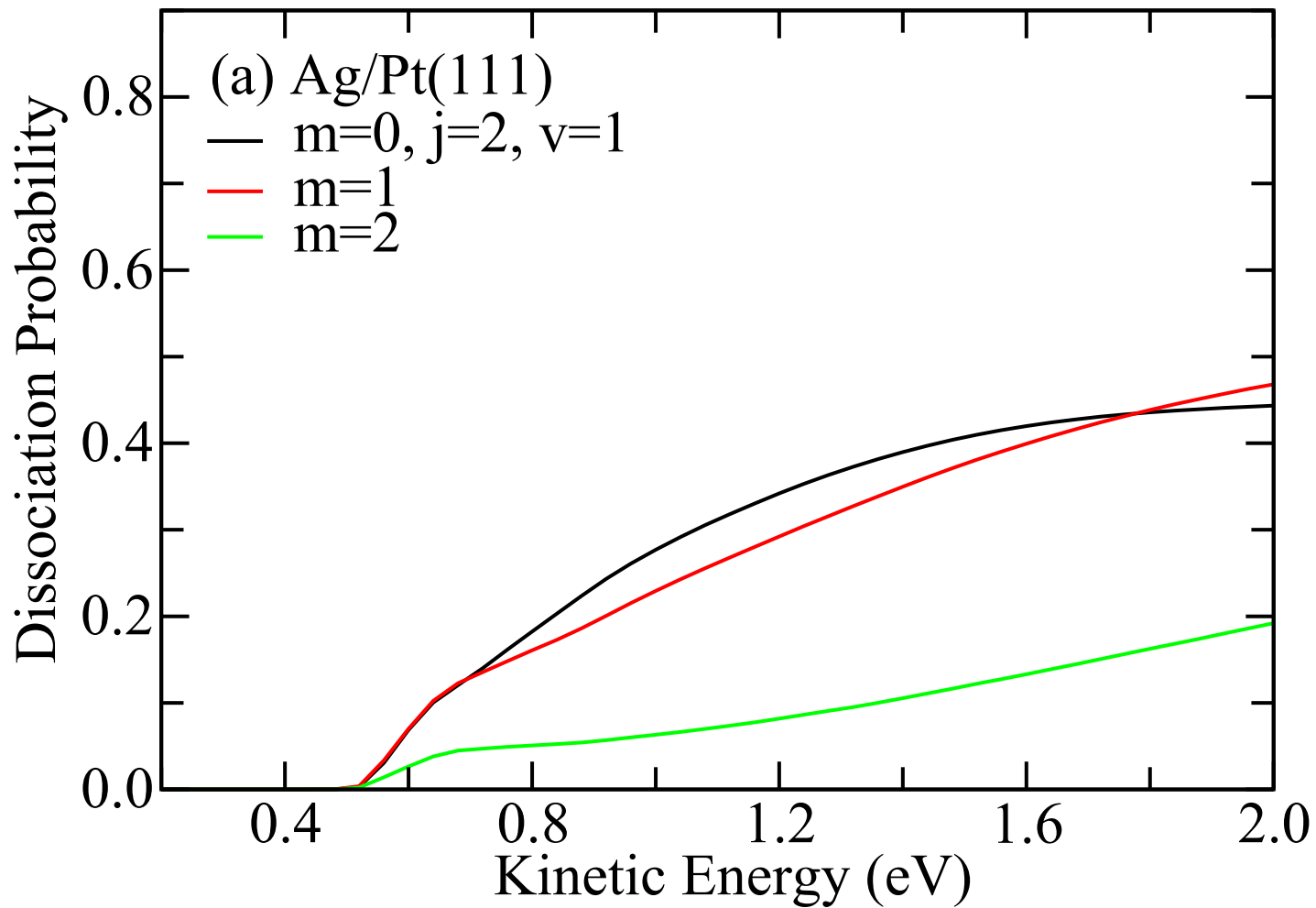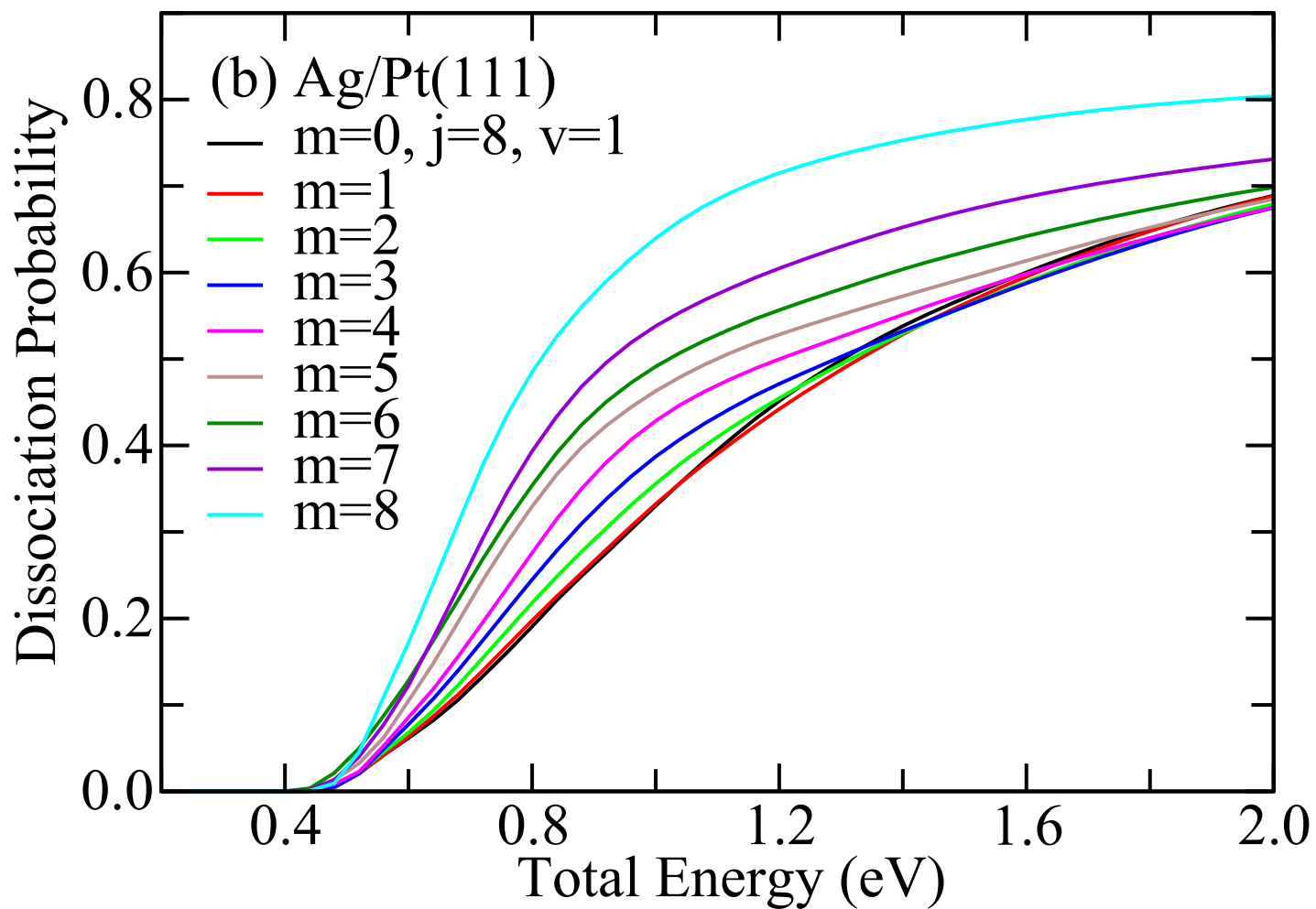

Supplement: SC-017-D6SC00201C-s002 [file SC-017-D6SC00201C-s002.zip › support/FigS7_AgPt_v1jm.pdf]
